# Recent Developments in Phenotypic and Molecular Diagnostic Methods for Antimicrobial Resistance Detection in *Staphylococcus aureus*: A Narrative Review

**DOI:** 10.3390/diagnostics12010208

**Published:** 2022-01-15

**Authors:** Andrea Sanchini

**Affiliations:** Freelance Trainer in Scientific Writing and Publishing, Hildegardstraße 15, 10715 Berlin, Germany; info@sanchini-writing.com

**Keywords:** *Staphylococcus aureus*, MRSA, antimicrobial susceptibility testing, rapid detection, antibiotic resistance, molecular methods, phenotypic methods, point of care, resistance detection, antimicrobial stewardship

## Abstract

*Staphylococcus aureus* is an opportunistic pathogen responsible for a wide range of infections in humans, such as skin and soft tissue infections, pneumonia, food poisoning or sepsis. Historically, *S. aureus* was able to rapidly adapt to anti-staphylococcal antibiotics and become resistant to several classes of antibiotics. Today, methicillin-resistant *S. aureus* (MRSA) is a multidrug-resistant pathogen and is one of the most common bacteria responsible for hospital-acquired infections and outbreaks, in community settings as well. The rapid and accurate diagnosis of antimicrobial resistance in *S. aureus* is crucial to the early initiation of directed antibiotic therapy and to improve clinical outcomes for patients. In this narrative review, I provide an overview of recent phenotypic and molecular diagnostic methods for antimicrobial resistance detection in *S. aureus*, with a particular focus on MRSA detection. I consider methods for resistance detection in both clinical samples and isolated *S. aureus* cultures, along with a brief discussion of the advantages and the challenges of implementing such methods in routine diagnostics.

## 1. Introduction

*Staphylococcus aureus* is a Gram-positive cocci-shaped bacterium that grows in the characteristic clusters of grapes. This bacterium encodes for both the staphylococcal protein A and the coagulase enzyme; these two factors have diagnostic importance since they are used to differentiate *S. aureus* from coagulase-negative staphylococci (CONS), which are usually less virulent. *S. aureus* commonly colonizes human skin and nasopharynx and it is estimated that around 15%–30% of healthy adults are nasal carriers of *S. aureus* [1,2]. However, *S. aureus* can also become an opportunistic pathogen, responsible for a wide range of clinical diseases, such as skin and soft tissue infections (impetigo, folliculitis or scalded skin syndrome), intravenous catheter-associated infections, food poisoning, toxic shock syndrome, osteomyelitis, pneumonia or bloodstream infections (BSI) [1]. 

Historically, *S. aureus* rapidly evolved and developed resistance to several classes of antibiotics. The resistance to penicillin, the first discovered beta-lactam antibiotic against *S. aureus* infection, was documented in 1942 [3]. Today, the vast majority of *S. aureus* isolates are resistant to penicillin [1]. To counteract this resistance, new semisynthetic beta-lactams antibiotics were developed (e.g., methicillin, oxacillin). In 1961, the first report of resistance to those new antibiotics was already published [4]. Today, methicillin-resistant *S. aureus* (MRSA) is one of the most common bacteria responsible for hospital-acquired infections and outbreaks [5]. In addition, MRSA has been isolated in community settings (where it can also cause outbreaks of skin and soft tissue infections or pneumonia in healthy adults [1,6,7,8]) and in livestock animals [9,10], and is characterized by multidrug resistance, being resistant, to varying degrees, to other antibiotics, such as macrolides, aminoglycosides, tetracyclines or fluoroquinolones [11]. Indeed, in 2017, the World Health Organization indicated MRSA as a high priority bacterium for which the development of new antibiotics is needed [12]. Lastly, since 1997 several studies have reported MRSA isolates with a reduced susceptibility (or fully resistance) to vancomycin, a glycopeptide antibiotic that is the drug of choice for therapy against serious MRSA infections [13,14].

Usually, the initial therapy of patients suspected to be infected with *S. aureus* is based on the experience of the treating physician; this empiric therapy includes the use of broad-spectrum antibiotics. When the results of pathogen identification and antimicrobial susceptibility tests (AST)s become available, usually in 24–72 h, the empirical therapy can be changed to a more targeted antibiotic therapy based on evidence from the specific case [15]. However, incorrect empirical therapy can have consequences such as increased lengths of stay in the hospital and intensive care unit (ICU), the emergence of drug resistance, antibiotic toxicity and increased costs for the patient and the healthcare system [16,17]. Therefore, the rapid and accurate detection of *S. aureus* and its drug resistance (ideally, shortly after or on the same day the patient sample is collected) is of clinical importance for the rapid de-escalation of therapy from broad-spectrum to targeted antibiotics [17].

In this narrative review, I provide an overview of recent phenotypic and molecular diagnostic methods for antimicrobial resistance detection in *S. aureus*. I will particularly focus on MRSA detection, since the differentiation between MRSA (which is often multidrug-resistant and, therefore, has limited treatment options) and methicillin-susceptible *S. aureus* (MSSA) isolates is of clinical relevance. I consider methods that can detect resistance on both clinical samples and isolated cultures, along with a brief discussion about their possible implementation in routine diagnostics. With this narrative review, I want to update the knowledge already discussed in the reviews from van Belkum in 2018 [18], Mizusawa in 2020 [19] and Buonomini in 2020 [20]. I hope this narrative review can help clinicians and microbiologists in choosing the proper diagnostic test and in interpreting the relative results. 

## 2. Methods

### 2.1. Search Strategy, Keywords and Databases

**Figure 1:**
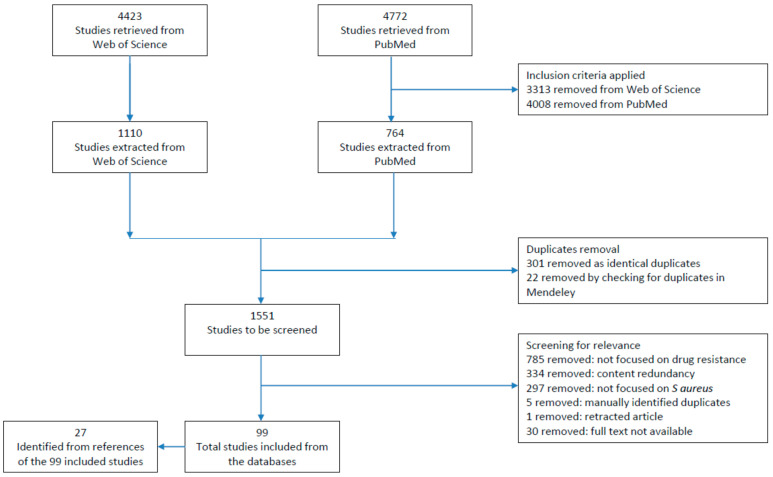
Overview of the search strategy and the study selection procedure used in this narrative review.

This narrative review was conducted following the guidelines published by Ferrari [21]. Four major topics relevant to the focus of this review were identified: phenotypic methods, molecular methods, antibiotic resistance detection and *S. aureus*. With these four concepts, a list of relevant keywords was developed. To help identify relevant keywords, the controlled vocabulary from the Medical Subject Headings (MeSH) of the National Library of Medicine (a thesaurus system) was used [22]. The databases Web of Science (WoS) Core Collection and PubMed were chosen, since both are human-curated databases [23]. In addition, WoS was chosen because of its large coverage [24], and PubMed was chosen because it includes the MeSH thesaurus-controlled vocabulary [22], making the search easier for users. Details about the search strategy and the study selection are shown in Figure 1. The following search string was used in WoS (13 October 2021): TS = (“resistance * detection *” OR “* microbial * susceptibility test *” OR “* microbial * sensitivity Test *” OR “point of care“ OR phenotypic drug resistance test * OR genotypic drug resistance test * OR resistance prediction * OR rapid detection * OR (sensitivity specificity AND “antibiotic * resistance *”)) AND TS = (“staphylococcus aureus”). The following search string was used in PubMed (13 October 2021): (resistance detection [Title/Abstract] OR antimicrobial susceptibility testing [Title/Abstract] OR antimicrobial sensitivity testing [Title/Abstract] OR “microbial sensitivity tests/methods” [Mesh] OR “microbial sensitivity tests/standards”[Mesh] OR point-of-care testing [MeSH Terms] OR point-of-care systems [MeSH Terms] OR phenotypic drug resistance testing OR genotypic drug resistance testing OR resistance prediction [Title/Abstract] OR rapid detection [Title/Abstract] OR (sensitivity and specificity [MeSH Terms] AND drug resistance [MeSH Terms])) AND staphylococcus aureus [Title/Abstract].

### 2.2. Inclusion Criteria and Duplicate Removal

The retrieved studies were then restricted to studies published in English in the period 2019–2021 to only focus on the most recent developments. The following types of documents were included: books, clinical trials, comments, editorials, guidelines, journal articles, letters, meta-analyses, reviews, systematic reviews, technical reports, proceeding papers, and early access. After applying those inclusion criteria, 1110 studies obtained from WoS and 764 studies obtained from PubMed were imported into Mendeley [25]. When merged into Mendeley, 323 studies were removed because of duplicates. A total of 1551 studies were screened for relevance by reading the title and the abstract. 

### 2.3. Screening for Relevance

Briefly, 785 studies were excluded because their main focus was not the detection of drug resistance. For example, studies were excluded if their focus was only on methods for *S. aureus* identification, on virulence factor characterization or on drug resistance in animal isolates (Figure 1). A total of 334 studies were excluded because of content redundancy; studies repetitively using the same methodologies or studies focused on the epidemiology of drug resistance or the molecular typing of isolates were excluded. An additional 297 studies were excluded because their main focus was not *S. aureus*; for example, if studies used *S. aureus* as a negative or a specificity control for a particular diagnostic technique. Finally, a total of 99 studies were included in this narrative review from the initial database search. In addition, 27 studies were included by a manual search in the reference list of the 99 selected studies. In Supplementary Material S1, a list of the 126 included studies is shown.

## 3. Phenotypic Methods for Antimicrobial Resistance Detection in *S. aureus*

Generally, in phenotypic methods, bacteria (*S. aureus*) need to grow in the presence of certain concentrations of the antibiotic to be tested. Using these methods, it is possible to evaluate the real phenotypic resistance. With some phenotypic methods it is possible to determine the exact antibiotic minimum inhibitory concentration (MIC)—the lowest concentration of antibiotic that inhibits bacterial growth—whereas in other methods, it is possible to determine only if bacteria are susceptible, intermediate or resistant to an antibiotic. Moreover, phenotypic methods can be divided into manual and (semi) automated. Phenotypic methods such as the broth microdilution method (BMD) or the disk diffusion method represent the classical and “gold standard” in AST [26]. Although phenotypic methods measure the real resistance and are widely used, they suffer from interlaboratory reproducibility issues; for example, factors affecting reproducibility include the operator conducting the test and manually interpreting/reading the results (a problem that can be solved using automated phenotypic methods), or the environmental/laboratory conditions such as growth media and reagents’ stability [27]. An overview of the phenotypic methods (and their performance) used by the studies selected for this narrative review is given in Table 1. 

### 3.1. Chromogenic Media

This is an example of a workflow using traditional culture-based phenotypic methods for antimicrobial resistance detection (or for MRSA detection): a clinical sample from a patient is streaked in a general growth media such as blood agar. After 16–24 h of incubation, a mixed culture (because of contaminants/colonizing bacteria) is often detected and *S. aureus,* therefore, has to be isolated and reinoculated in a fresh plate, i.e., a subculture, for an additional 16–24 h. When *S. aureus* is isolated in the subculture, an AST test can be performed (for another 16–24 h) and an MRSA, for example, can be detected. This process can take up to 72 h [44]. To reduce this turnaround time (TAT) (i.e., time from sample collection to results), chromogenic media have been developed. Chromogenic media are selective media based on selective supplements (inhibiting the growth of other bacteria) and cefoxitin or oxacillin (inhibiting MSSA growth). Although the nomenclature “methicillin resistance” is universally used, methicillin is not tested anymore; oxacillin, and especially cefoxitin, are the preferred drugs due to better stability and clearer endpoints [45,46]. Chromogenic media can identify MRSA by colonies’ color reactions in 18–26 h in the first plate streaked with the clinical sample (e.g., nasal swabs or wound swabs) [44,47]. Madhavan and colleagues used a new chromogenic media (CHROMagar, HiCrome ™ Rapid MRSA Agar Plate-MP1974, HiMedia, New Delhi, India) to determine the methicillin resistance in 100 *S. aureus* subcultures originally isolated from different clinical samples. Using gold-standard methods, 72 MRSA and 28 MSSA were identified and the sensitivity and specificity of the chromogenic media for MRSA detection were 100% and 78.6%, respectively. Six false-positive MRSA were identified by the chromogenic media after 24 and 48 h of incubation; those six isolates were confirmed to be MSSA using other phenotypic methods [28]. In another study, the authors investigated 48 *S. aureus* subcultures to determine their methicillin resistance using chromogenic MRSA (RTA Laboratories, Kocaeli, Turkey). They identified 19 MRSA and 29 MSSA and the sensitivity and specificity of the chromogenic media for MRSA detection after 24 h of incubation were 78.9% and 41.3%, respectively; the specificity improved to 58.6% if the incubation was extended to 48 h [29]. Chromogenic media represent an easy and low-cost way to screen for MRSA carriers, but usually do not detect MSSA, which is relevant clinical information for the proper antibiotic choice.

### 3.2. Broth Microdilution Method (BMD)

In the BMD method, bacteria from an isolated culture are inoculated in liquid media with different antibiotic concentrations (in two-fold serial dilutions) in 96-well plates and incubated for 16–24 h. Turbidity in the wells indicates bacterial growth in the presence of the specific antibiotic concentration; therefore, the antibiotic MIC can be determined according to the Clinical and Laboratory Standards Institute (CLSI) and the European Committee on Antimicrobial Susceptibility Testing (EUCAST) criteria [45,46,48]. The BMD method is one of the gold-standard phenotypic methods and is low-cost, but it requires an isolated culture and an overnight incubation. To reduce the TAT of the BMD, Mahmoud et al. used a rapid and colorimetric BMD method that can provide AST results in 7 h. Two dyes, 3-(4,5 dimethyl thiazole-2-yl)-2, 5 diphenyl tetrazolium bromide (MTT) and 2,3,5-triphenyl tetrazolium chloride (TTC) were used as rapid indicators of oxidation-reduction. The authors analyzed 40 *S. aureus* and 40 CONS clinical isolates. In the rapid BMD, the 96-well plates were incubated for only 6 h (instead of 16–24 h in the standard BMD), and then MTT (or TTC) was added and plates were re-incubated for one hour. After 7 h, the final reading was carried out. The authors compared the rapid BMD with the disk diffusion and the standard BMD. The rapid BMD using MTT gave the best performance in terms of categorical agreement (CA, the percentage of isolates that belong to the same susceptibility group) in *S. aureus* isolates: 100% for gentamycin and erythromycin, 97.5% for oxacillin, 95.0% for tetracycline, 87.5% for ciprofloxacin and 72.5% for clindamycin. The rapid BMD correctly identified 21/21 MRSA isolates (sensitivity: 100%) and 18/19 MSSA isolates (specificity: 94.7%). The rapid BMD is easy to interpret, provides accurate AST results and is low cost. Therefore, it represents a good method that could be routinely implemented, especially in settings with limited resources [30].

### 3.3. Disk Diffusion Method

In the disk diffusion method, colonies from an isolated bacterial culture are suspended in growth media until a concentration of ca. 1–2 × 10^8^ colony-forming unit (CFU)/mL (equivalent to the 0.5 McFarland turbidity unit). This bacterial suspension is then inoculated in cation-adjusted Mueller-Hinton (MH) agar plates, and paper disks containing fixed antibiotic concentrations are applied to the agar surface. After 16–24 h of incubation, the diameter of the growth inhibition of each disk is measured. The size of the diameters is used to interpret whether bacteria are susceptible or resistant to the antibiotic according to CLSI or EUCAST criteria [45,46,48]. The disk diffusion method provides a categorical assignment (susceptible or resistant) but not the exact MIC value [49,50]. To test methicillin-resistance, CLSI and EUCAST recommend the use of the cefoxitin disk (30 µg) [45,46]. In their study, Madhavan and colleagues reported a sensitivity of 97.2% (70/72) and a specificity of 100% of the cefoxitin disk diffusion method for MRSA detection. The two false-negative MSSA identified had an inhibition zone diameter of 23–24 mm, close to the 22mm breakpoint [45,46]. Indeed, both those false negatives were detected as MRSA by VITEK 2 and chromogenic agar. The author suggested that in the case of a borderline inhibition zone diameter, a second confirmatory test might be necessary [28]. The disk diffusion method is a gold-standard method, and is accurate, reliable, easy to perform and low cost. However, it needs an isolated culture followed by overnight incubation. To reduce this TAT, several options were reported in the literature. For example, Pilmis and colleagues performed the disk diffusion method on MH rapid agar MHR-SIR (i2a, France) directly on flagged-positive blood culture bottles (hereafter referred to as positive blood cultures) with 6–8 h of incubation and evaluated the clinical impact of this method in their hospital. The reading of the inhibition zone diameters was carried out automatically with the SIRscan ^®^ 2000 Automatic system (i2a, France). Out of the 330 patients with BSI analyzed, 29 were MRSA and 50 were MSSA. Two groups of patients were compared: patients who were managed by using the results of the MHR-SIR method versus patients who were managed by using the results of conventional methods. The author found that the MHR-SIR method allowed for the better de-escalation of antibiotic treatment compared to the conventional methods [32]. In a previous study from the same group, the authors compared the rapid MHR-SIR with the traditional disk diffusion method and obtained 315 CA (97.8%), 6 (1.9%) minor errors (mE) and 1 (0.3%) very major error (VME) in 23 *S. aureus* isolates (4 MRSA and 19 MSSA) from positive blood cultures [31]. A VME occurs when the reference method reports resistance but the test method reports susceptibility. A major error (ME) is the opposite scenario; a mE occurs when one method reports intermediate resistance but the other reports either susceptibility or resistance [51]. 

Another attempt to reduce the TAT is exemplified by the work of Åkerlund and colleagues. They evaluated the EUCAST rapid AST (RAST) disk diffusion method directly from positive blood cultures, and plates were incubated for 4, 6 and 8 h instead of overnight incubation. After this short incubation, AST results were interpreted according to EUCAST preliminary breakpoints [52]. To limit false-resistant or false-susceptible results, the EUCAST introduced the area of technical uncertainty (ATU), a sort of buffer area where the results cannot be interpreted and might require either a longer incubation time or a confirmatory test. Among the 337 *S. aureus* analyzed, 24 were identified as MRSA after 4, 6 and 8 h of incubation with 100% sensitivity. The RAST disk diffusion could replace the traditional disk diffusion for a more rapid AST of positive blood cultures. However, not all new breakpoints for rapid AST are available and the interpretation of the results might be more challenging, since after a few hours of incubation, bacterial growth is low and the zone diameter margins are less demarcated. A more automated interpretation of the results might overcome this limitation [33]. Jasuja and colleagues also applied the RAST in positive blood cultures inoculated with sterile body fluids. Among the 197 *S. aureus* identified, 14 were MRSA (100% sensitivity, 99.4% specificity). The authors should have probably compared the RAST method with a more similar AST method (e.g., BMD or standard disk diffusion) and not with the VITEK 2 [34]. In a similar study from the same group, the authors used the rapid disk diffusion method on positive blood cultures and calculated that the TAT was 17.5 h shorter [35]. 

### 3.4. Gradient Diffusion Methods (Etest)

In the gradient diffusion methods, gradient concentrations of the antibiotic are impregnated in a plastic strip that, similar to disk diffusion, can be placed on MH agar to determine the antibiotic MIC [50]. The Etest (bioMérieux AB BIODISK) is an example of a commercial gradient diffusion test. The Etest is not used to determine methicillin resistance in *S. aureus,* since this test is more expensive than the cefoxitin disk diffusion method. However, the Etest is often used to determine the MIC to vancomycin. The CLSI defined an *S. aureus* isolate susceptible to vancomycin if the MIC is ≤2 μg/mL, intermediate (vancomycin-intermediate *S. aureus*, VISA) if the MIC is 4–8 μg/mL and resistant if the MIC is ≥16 μg/mL [45]. In addition, in heterogeneous VISA (hVISA) isolates, the vancomycin MIC is within the susceptible range when tested with standard methods, but a proportion of cells/the population is resistant to vancomycin [53]. Kuo and colleagues investigated the MICs of vancomycin in 216 MRSA isolates from blood cultures using the Etest, the BMD and the VITEK 2. The authors focused on isolates with a vancomycin MIC ≥ 2 in MRSA since this is the value at which the Infectious Diseases Society of America recommends using antibiotics other than vancomycin to treat MRSA infections. Fifteen MRSA isolates had a vancomycin MIC ≥ 2. The Etest underestimated the proportion of isolates with a MIC ≥ 2, since it identified two false-negative results [37]. For an overview of the epidemiology and AST of vancomycin in *S. aureus*, readers can consult the review of Wu [54].

### 3.5. Automated Commercial Phenotypic Methods

Phenotypic AST in *S. aureus* (and in other bacteria) can also be performed through automated methods/systems. The advantages of using those systems over manual phenotypic ASTs are results standardization, shorter TAT (4.5–24 h) and the (semi) automation of the procedure. Disadvantages could be that the range of tested antibiotics is often fixed (incorporated in cartridges/panels) or that only a few drug concentrations can be tested. The most commonly Food and Drug Administration (FDA)-approved systems for automated AST used in diagnostics are VITEK 2 (BioMérieux, Inc., Durham, NC, USA), MicroScan (Beckman Coulter, Renton, WA, USA), Phoenix (BD Diagnostics, Sparks, MD, USA) and Sensititre (Thermo Fisher Scientific, Oakwood Village, OH, USA) [50]. All those methods have automated systems to detect bacterial growth in the presence of specific antibiotic concentrations [32,50]. For a detailed description of the four methods, please refer to Jorgensen (2009) [50]. Madhavan and colleagues reported a sensitivity of 97.2% and a specificity of 100% for the MRSA detection using the VITEK 2. They identified 2 false-negative MSSA out of the 72 MRSA identified. One limitation of the study is that the authors compared the performance of a phenotypic test (VITEK 2) with a molecular test (*mecA*-PCR, see below), instead of comparing the VITEK 2 with a similar phenotypic method such as the BMD [28]. 

Considering the resistance to antibiotics other than methicillin, Yoo and colleagues retrospectively analyzed the VITEK 2 results of 22.067 MRSA. Out of the 27 isolates resistant to linezolid (MIC ≥ 8 μg/mL) identified by VITEK 2, only 4 were confirmed linezolid-resistant by the reference BMD method, resulting in 23/27 (85.2%) MEs. The authors observed a high rate of false-resistance results for linezolid using VITEK 2. Therefore, in the case of linezolid resistance isolates detected using VITEK 2, a confirmatory test might be needed [36]. In another study, the authors found that the VITEK 2 overestimated the proportion of MRSA isolates with a vancomycin MIC ≥ 2. Using BMD as a reference method, 15 MRSA had a vancomycin MIC ≥ 2, whereas the VITEK 2 identified 28 MRSA with this MIC, resulting in 13 false-positive results. The authors concluded that in the case of a vancomycin MIC ≥ 2 identified with the VITEK 2, a second confirmatory test should be performed before shifting to second-line antibiotics, to reduce inappropriate therapy and resistance emerging [37]. Al Rawahi and co-authors identified discrepancies in the susceptibility of *S. aureus* to trimethoprim/sulfamethoxazole (SXT) by using the automated BD Phoenix (BD Diagnostic Systems, Sparks, MD, USA) and the disk diffusion method. In the 642 *S. aureus* analyzed, the disk diffusion test detected 636 susceptible, 2 intermediate and 4 resistant isolates to SXT. On the other hand, the Phoenix identified 586 susceptible and 50 resistant isolates resulting in a CA of 91.9%, with 50 (7.9%) MEs and 2 (0.3%) mE. The specificity of the Phoenix BD system to detect the resistance to SXT was 91.3% (586/642). The authors restricted the analysis only to MRSA and found that the CA for the SXT resistance testing was significantly lower (82.9%) compared to MSSA, highlighting a problem due to the low therapeutic options to treat MRSA infections. The study also highlighted the need to monitor the performance of the Phoenix BD system regarding SXT resistance determination, maybe by performing a confirmatory test in the case of an MRSA infection [38]. 

Cherkaoui and colleagues evaluated the performances of the Copan WASP Colibri Coupled to the Radian In-Line Carousel and Expert System, a fully automated disk diffusion method, and compared the results with the VITEK 2. Among the 107 *S. aureus* isolated from various clinical samples, no discordant results were observed in the resistance to cefoxitin, gentamicin, clindamycin, erythromycin, fusidic acid, rifampicin, linezolid, or tigecycline. The overall CA between was 99.9%. Although the VITEK 2 still requires some manual work, the Copan has the following advantages: it is fully automated from the preparation of the inocula until the interpretation of the results, it is flexible since the antibiotic disks can be easily replaced with others, it is more reliable in detecting heteroresistance (as the traditional disk diffusion method) and it allows for the detection of mixed culture. One limitation of the study could be that the Copan should have been compared with more similar methods rather than with the VITEK 2 [39]. In the same group, the authors evaluated the performance of an earlier semiautomated version of the Copan, reaching a similar performance [51]. 

The Accelerate PhenoTest ™ BC is another automated system recently approved by the FDA. It performs the identification and AST of both Gram-positive and -negative bacteria directly from positive blood cultures. The system automatically purifies blood cultures from impurities. Subsequently, bacterial cells are immobilized and identification is achieved microscopically through single-cell analysis. Single-cell identification is performed using fluorescent in situ hybridization (FISH) with specific bacterial probes. Then, samples grow in MH agar with the specific antibiotic. The system provides identification in 90 min and AST in 7 h and it also indicates whether samples contain only one pathogen or not (“monomicrobial call”). Pancholi and co-authors evaluated the performance of the Accelerate system in 247 *S. aureus* and reported a sensitivity and specificity for *S. aureus* detection of 98.0% and 98.9%, respectively. The Accelerate system reached 99.5% CA for *S. aureus* and methicillin resistance, with one ME. The sensitivity and specificity for MRSA detection were 100% and 98.8%, respectively [17]. Lutgring and coauthors evaluated the performance of the Accelerate PhenoTest ™ BC and found 100% sensitivity and specificity for MRSA detection [40]. A notable advantage of the Accelerate system is that it can perform identification and AST together, without the need for prior identification with another method. 

Sanchez-Carrillo and Boland evaluated the Alfred AST^®^ system for rapid AST directly on positive blood cultures. This system detects bacterial growth by turbidimetry using a light-scattering technology. The antibiotic panels are customizable; however, the system needs a prior identification method to be assigned to the proper antibiotic panel. Both studies detected a CA of 100% when analyzing 7 and 30 *S. aureus* isolates; however, it is not clear how many *S. aureus* were MRSA or resistant to other antibiotics. Therefore, larger studies testing the system with more resistant *S. aureus* are needed [55,56].

### 3.6. Penicillin-Binding Protein (PBP) 2A Detection Assay

The PBP2a is encoded by the *mecA* gene and is responsible for the resistance to methicillin (or oxacillin, or cefoxitin) in *S. aureus*. Therefore, another phenotypic method to detect MRSA is the detection of the PBP2a protein directly on isolated *S. aureus* colonies. Two major commercial assays exist: the PBP2a latex agglutination assay (Oxoid, Thermo Fisher Scientific Inc., Lenexa, KS, USA) and the Alere PBP2a SA culture colony assay (Abbott Diagnostics, Abbott Park, IL, USA), an immunochromatographic method [44]. Khawaja and colleagues used the PBP2a latex agglutination assay in 105 clinical MRSA isolates and reported a sensitivity of 98.9% and a specificity of 77.8% [41]. 

The resistance to oxacillin is mediated not only by *mecA* but also by the *mecC* gene (originally named *mecA*_LGA251_), which shares 70% nucleotide homology with *mecA* and encodes for the PBP2c, complicating the diagnostic tests based on *mecA*-PCR and PBP2a detection [57,58]. Dupiex and colleagues evaluated the performance of the last version of the immunochromatographic assay developed by the Alere PBP2a SA culture colony test (SACCT) (Alere, Scarborough, ME). They analyzed 73 MRSA (63 *mecA* positive and 10 *mecC* positive), 10 MSSA and 10 CONS. They also evaluated the PBP2a SA culture colony test performance after PBP2a/PBP2c induction by growing isolates in the presence of the cefoxitin disk (30 µg). The method revealed 100% sensitivity for the PBP2a detection but it cannot detect the PBP2c, even after cefoxitin induction. Therefore, no improvements were observed in the detection of the PBP2c between the current and the previous version of the PBP2a SA culture colony test (SACCT). Therefore, a negative result with the PBP2a SACCT does not imply methicillin susceptibility, since the isolate can be a *mecC*-positive MRSA [42]. 

Kolesnik-Goldmann also used the PBP2a SACCT but they modified the protocol. They tested the SACCT on the shortly incubated (4–6 h instead of 18–24 h) staphylococcal subcultures (induced or not induced with cefoxitin) of positive blood cultures. A total of 38 *S. aureus* subcultures were analyzed, with 25 *mecA*-positive MRSA and 13 MSSA. The rapid SACCT reported a sensitivity of 96.0% (24/25) and a specificity of 92% (12/13). If subcultures were previously induced with cefoxitin, 100% sensitivity and specificity were reached. The authors observed that reading the final results after 10 min (instead of 5 min) could generate clearer bands [43]. 

### 3.7. Matrix-Assisted Laser Desorption/Ionization Time-of-Flight Mass Spectrometry (MALDI-TOF MS)

In recent years, mass spectrometry entered clinical microbiology laboratories. The MALDI-TOF system detects the proteins present in bacteria grown on a solid medium. Bacterial colonies are fixed in a crystalline matrix and are then bombarded by laser. The sample’s proteins are ionized and then accelerated to the ion detector, which measures protein charges and time of impact. Lower-mass proteins will arrive sooner than higher-mass proteins. Using a reference and known mass, the mass of the proteins examined can be determined and is visualized into a mass spectrum, where each peak of the spectrum on time corresponds to the specific mass of a protein. Therefore, one microorganism will have a specific mass spectrum corresponding to its protein profile, and this mass spectrum can be used for bacterial identification. The application of MALDI-TOF for pathogen identification in clinical laboratories is widespread. The two most common and FDA-approved MALDI-TOF systems are the VITEK MS (bioMérieux Inc., Durham, NC, USA) and the MALDI Biotyper CA System (Bruker Daltonics, Billerica, MA, USA) [44]. On the contrary, the application of MALDI-TOF for routine AST is still a developing field. By using MALDI-TOF, several means exist to determine resistance or susceptibility to an antibiotic, e.g., the detection of proteins conferring resistance, the detection of a mass peak (biomarker) associated with resistance or antibiotic degradation. For reviews regarding the possibilities that MALDI-TOF offers in AST, please refer to these publications [59,60,61,62,63]. One notable advantage of the MALDI-TOF is that it could perform both identification and AST, simplifying the diagnostic workflow without the need to have separate methods. In addition, MALDI-TOF is fast (few minutes once an isolated culture is available but can also be performed on positive blood culture, see below), with a relatively cheap cost per isolate and is easy to perform. The challenges are the initial high cost of the MALDI-TOF instrument and the inter-laboratory reproducibility, since MALDI-TOF does not consistently yield the same intensities or peak patterns and, therefore, there is a need for various and larger databases of specific peaks for MRSA [60]. Several studies used MALDI-TOF in particular to discriminate between MRSA and MSSA isolates. An overview of those studies is presented in Table 2.

Kim and colleagues used MALDI-TOF on clinical isolates to identify peaks or biomarkers that could differentiate MRSA from MSSA. By analyzing a database set of 320 *S. aureus* isolates, the authors identified 13 peaks specific for MRSA and 8 peaks specific for MSSA isolates. Based on this information, the authors developed an algorithm based on 21 peaks to discriminate between MRSA and MSSA. They applied the algorithm to 181 isolated *S. aureus* and it could detect MRSA with a sensitivity of 87.6% and a specificity of 71.4% [64]. Paskova et al. evaluated the detection of the peptide phenol soluble modulin (PSM-mec) and δ-toxin peaks (specific for MRSA and especially with those containing the SCC*mec* types II, III and VIII) as a method to discriminate MRSA from MSSA. By analyzing 35 MRSA, a sensitivity of 70% (14/20) was reached for MRSA containing SCC*mec* II or III, but 0/15 MRSA containing the SCC*mec* I, II and V were correctly identified, highlighting the poor discriminatory power of these peaks [65]. Hu and colleagues evaluated the discriminatory power of the PSM-mec peak in 241 MRSA and 106 MSSA and found a sensitivity of 60.2% and specificity of 100%, but no information about the SCC*mec* type of the isolates was available [66]. Another attempt to identify through MALDI-TOF-specific MRSA peaks was conducted by Flores-Treviño and colleagues. They analyzed 36 MRSA and 31 MSSA and found a peak with a m/z of 4594 that could discriminate MRSA isolates (where the peak is present as a duplet) from MSSA isolates (where the peak is a singlet). Using this peak to detect MRSA isolates, a sensitivity of 83.3% and a specificity of 96.8% were reached. The peak seems to be connected with the 50S ribosomal subunit and could be further investigated as a biomarker for MRSA [67]. Liu applied a machine learning approach (support vector machine algorithm) to identify peaks that could discriminate between MRSA and MSSA isolates They analyzed 194 MRSA and 258 MSSA and found 38 features that could discriminate MRSA from MSSA with a sensitivity of 84.0% and a specificity of 88.0% [68]. For an overview of studies on the identification of specific MRSA peaks, please refer to the works of Burckhardt and Liu [15,68].

Idelevich developed a MALDI-TOF-based direct-on-target microdroplet growth assay (DOT-MGA); microdroplets of bacterial suspension are first incubated in broth, and then inoculated on MALDI-TOF, with and without an antibiotic. After broth removal, growth in the presence of the specific antibiotic is measured [71]. Nix and co-authors evaluated the MALDI-TOF-based DOT-MGA directly on positive blood cultures to detect MRSA isolates. A total of 14 MRSA and 14 MSSA clinical isolates were included, plus 16 control MRSA isolates representing the SCC*mec* types from I to V. Once a blood culture bottle was flagged positive, the authors tried three different protocols: filtration/dilution, lysis/centrifugation and differential centrifugation. Then, the inoculum from the three different protocols was used for AST through DOT-MGA with 4, 5 and 6 h of incubation. Finally, the three inocula were added to the MALDI target, with and without cefoxitin. The lysis/centrifugation protocol gave the best performance, reaching 100% sensitivity and specificity for MRSA after 4, 5 or 6 h of incubation. This rapid AST method can be integrated in a routine diagnostic workflow for blood cultures, since it can perform AST on the same working shift (or day) as the blood culture became positive [69]. Horseman and colleagues also used the DOT-MGA, but with the VITEK MS (bioMérieux, Durham, NC, USA) on isolated cultures of 20 MRSA and 20 MSSA and obtained 100% sensitivity and specificity for MRSA detection [70].

## 4. Molecular Methods for Antimicrobial Resistance Detection in *S. aureus*

Molecular methods diagnose antimicrobial resistance by detecting resistance-associated genes. One of the major advantages of using molecular methods is the reduced TAT compared to phenotypic methods, since there is no need to wait for bacterial growth in the presence of antibiotics. In addition, molecular methods are less affected by those reproducibility issues typical of phenotypic methods (see previous section). However, molecular methods also have limitations: they do not detect the real phenotypic resistance behaviors, do not determine the MIC, can detect only known genes responsible for resistance and, therefore, cannot detect new resistance or genes [17,72]. Moreover, sometimes bacteria possess the resistance gene but are still susceptible to the antibiotic (see Section 4.1). In *S. aureus*, the resistance to methicillin (oxacillin, cefoxitin) is mediated by the *mecA* gene which encodes for the PBP2a. The *mecA*-PCR is a gold-standard molecular method to detect MRSA. The *mecA* gene is included in a mobile genetic element called staphylococcal cassette chromosome *mec* (SCC*mec*), together with regulatory genes (*MecI/MecR1/MecR2*), *ccr* genes responsible for mobility and other antibiotic resistance genes. The SCC*mec* element is inserted in the *orfX* locus of the *S. aureus* genome. Currently, 14 SCC*mec* types have been identified. The SCC*mec* typing is mostly useful for molecular surveillance rather than for clinical utility [19,73]. As said in Section 3.6, the *mecA* is not the only gene responsible for methicillin resistance; *mecC*, the plasmid-carried *mecB* [74] and *mecD* [75], exist, although the latter two have rarely been identified. An overview of the molecular methods (and their performance) used by the studies selected for this narrative review is given in Table 3.

### 4.1. Detection of Oxacillin-Susceptible MRSA (OS-MRSA)

OS-MRSA are phenotypically susceptible to oxacillin-cefoxitin but are *mecA*-positive [88]. From a diagnostic perspective, these OS-MRSA isolates can be considered a mistake of the phenotypic methods that can be corrected with molecular methods. Using the BMD method, an oxacillin MIC ≥ 4 µg/mL (or cefoxitin MIC ≥ 8 µg/mL) indicates the presence of an MRSA [45]. A typical OS-MRSA isolate is *mecA*-positive, but has an MIC for oxacillin of 2 µg/mL, so it would be wrongly identified as MSSA, if only a phenotypic test is performed. Liu and colleagues identified 17 OS-MRSA among 377 (4.5%) clinical *mecA* positive MRSA. The authors observed that the VITEK 2 system is problematic in detecting OS-MRSA isolates, although an additional cefoxitin screen is included. The VITEK 2 could correctly detect 6/17 OS-MRSA stains. A better performance was reached using the cefoxitin disk diffusion test, where 15/17 OS-MRSA were correctly identified. Therefore, the cefoxitin disk diffusion test might be needed to additionally test *S. aureus* isolates with a borderline oxacillin MIC (1–2 µg/mL) identified with automated AST systems. The authors also observed hetero-resistance to oxacillin since a high oxacillin resistance could be induced in all the identified OS-MRSA after in vitro exposure to growing concentrations of oxacillin. The authors recommend that clinicians treat those “cryptic” OS-MRSA with antibiotics normally used for MRSA, such as linezolid or vancomycin, rather than beta-lactams antibiotics [89].

Liu and colleagues identified 14 OS-MRSA isolates in 1200 clinical *S. aureus*. All of the 14 *mecA*-positive isolates were MSSA according to the BMD method, VITEK 2 and BD Phoenix 100. The cefoxitin disk diffusion test correctly detected 3/14 isolates as MRSA, whereas the PBP2a agglutination assay correctly identified 6/14 MRSA. To manage OS-MRSA infections, the authors recommend performing the PCR for the *mecA* and *mecC* genes in the case of patients suffering from severe *S. aureus* infections who are receiving beta-lactams antibiotics due to assumed MSSA infections determined with phenotypic methods, especially if the course of the disease is unfavorable. If an OS-MRSA is wrongly identified as MSSA and beta-lactam antibiotics are given, the oxacillin resistance subpopulation will be selected and will be responsible for treatment failure [90]. In another study, 11 OS-MRSA isolates were isolated from ocular infections [91]. Boonsiri et al. analyzed 43 OS-MRSA clinical isolates and found that cefoxitin performed better (19/43 isolates correctly identified as MRSA) than the automated system or the Etest in managing OS-MRSA, but was still not optimal, so a confirmatory PCR might be required [92]. For studies investigating the genetic basis of OS-MRSA, please consult [88,92,93,94]. 

### 4.2. Automated/Semiautomated Commercial Molecular Methods

#### 4.2.1. Cepheid GeneXpert ^®^

The GeneXpert system can detect MRSA directly in several clinical samples through real-time PCR targeting the *mecA/C* genes, the *orfX*-SCC*mec* junction and the *spa* gene (encoding for the *S. aureus*-specific staphylococcal protein A). All the PCR steps are automated and take place in disposable cartridges [44]. Dewar evaluated the Xpert SA Nasal Complete, which is validated for screening. Among 605 nasal samples, 17 samples were identified as MRSA by GeneXpert, with seven false positives. The GeneXpert sensitivity for MRSA detection was 100% and the specificity was 98.8% [76]. Ayebare and colleagues used the Xpert SA Nasal Complete (and other methods such as chromogenic media and Hain GenoQuick MRSA) to assess the prevalence of MRSA nasal carriage in their hospital. The authors created a composite reference standard, meaning that a sample was considered positive if tested positive by at least one of the three methods. From the 500 nasal swabs screened, 27 were MRSA. The sensitivity and specificity of the Xpert SA Nasal Complete were 51.8% (14/27) and 100%, respectively. By not having a standard reference culture to compare with, it is difficult to assess whether any susceptible isolate was erroneously identified as MRSA (false positive) [77].

The Xpert MRSA/SA blood culture (BC) assay (Cepheid) can be performed on positive blood cultures. McHugh et al. analyzed 264 positive blood cultures with Gram-positive cocci and detected 1 MRSA and 38 MSSA and reported a complete agreement between the GeneXpert system and the reference method. Using the Xpert MRSA/SA BC, the TAT was 1.7 h compared to 25.2 h with conventional methods. In addition, in 40/238 of analyzed patients (16.8%), the use of Xpert MRSA/SA BC results allowed for early changes in patient management; for example, patients started a specific *S. aureus* therapy a day earlier (than with the results of traditional methods), patients changed their antimicrobial prescription due to a positive Xpert result or patients interrupted therapy due to a negative Xpert result (and absence of clinical symptoms) [78]. In another study, the authors retrospectively investigated the impact of the Xpert MRSA/SA BC on time to optimal therapy and therapy duration in 101 positive blood cultures from pediatric patients. The pediatric patients were divided into two groups: patients managed according to the results of traditional AST (*n* = 61) versus patients managed according to the results of Xpert MRSA/SA BC (*n* = 40). Using Xpert MRSA/SA BC, the time to optimal therapy was around 20 h shorter, decreasing from 61.5 to 42.5 h from the blood culture collection. Moreover, MSSA-infected patients were de-escalated to a more direct therapy sooner, with the total hours of vancomycin therapy decreasing from 48.1 to 25.8 h [79]. 

Titecat and colleagues reviewed the studies evaluating the Xpert MRSA/SA SSTI on joint aspirations, tissue or bone specimens, from bone and joint infections [95]. Paonessa and colleagues used the Xpert MRSA/SA SSTI (validated for the diagnosis of skin and soft tissue infections) on 247 bronchoalveolar lavage (BAL) samples and the Xpert MRSA/SA SSTI reached a sensitivity of 95.7% and specificity of 98.2% for MRSA detection. The authors conducted a pilot clinical trial to evaluate if direct MRSA testing on BAL samples can safely reduce the use of anti-MRSA antibiotics (linezolid, vancomycin) in mechanically ventilated patients with suspected MRSA pneumonia. The suspected pneumonia patients were divided into two groups: patients managed according to the results of traditional AST (*n* = 23) versus patients managed according to the results of the Xpert MRSA/SA SSTI (*n* = 22). Using Xpert MRSA/SA SSTI, the time of linezolid/vancomycin treatment for initially suspected pneumonia patients was about 40 h shorter, decreasing from 72 to 32 h. Moreover, the time of anti-MRSA antibiotic administration in the following 28 days was about 70 h shorter, decreasing from 122 to 46 h. In 9/22 patients, the negative result of the rapid Xpert MRSA/SA SSTI (on Gram-positive cocci) prompted the safe interruption of vancomycin/linezolid treatment, with less adverse effect for patients, less development of antibiotic resistance, and more resources and time saved for the healthcare system [80]. Such studies quantify the clinical impact of the GeneXpert system. Coppens used the Xpert MRSA/SA ETA, an assay only for research, on endotracheal aspirates. A total of 79 endotracheal aspirates were analyzed and 1 MRSA and 40 MSSA were identified (100% sensitivity and specificity) [96].

#### 4.2.2. Hologic Panther Fusion ^®^ MRSA

Maurin and coauthors evaluated the performances of the Hologic Panther Fusion ^®^ MRSA to screen for MSSA or MRSA nasal carriage and compared the result of this assay with chromogenic media results. The Panther Fusion ^®^ MRSA can discriminate between MSSA and MRSA by detecting *mecA/C*, *gap* gene (*S. aureus* specific) and the SCC*mec*-*orfX* junction region. The method is fully automated and based on PCR and Invader chemistries [97]. The authors analyzed 434 nasal samples from hospitalized adults. Compared to chromogenic media, the Panther Fusion ^®^ reached an initial 88.0% CA. The authors further analyzed discordant results using Xpert SA Nasal Complete or broth enrichment. Thirty isolates were identified as MSSA using the Panther Fusion ^®^ but were negative on chromogenic media culture. Additional tests confirmed that 25 of those 30 were MSSA. In addition, nine samples were identified as MRSA using Panther Fusion ^®^ but were negative on chromogenic media culture. Additional tests confirmed that five of those nine were MRSA. That evidence pointed out that chromogenic culture had a lower sensitivity compared to Panther Fusion ^®^ for MSSA and MRSA detection, but the choice of chromogenic media as a reference method at least, at first sight, might have been wrong, even if the authors wanted to compare two screening methods, such as chromogenic media and the Panther Fusion ^®^. Maybe the method should be compared with a more similar method (e.g., GenXpert, *mecA*/*mecC*-PCR). After the analyses of discrepant results and the correction of the gold standard, 30 MRSA and 112 MSSA were detected and the Panther Fusion ^®^ MRSA reached 97.9% CA for MRSA detection [72].

#### 4.2.3. MRSA/SA ELITe MGB Assay

Boattini and colleagues evaluated the performance of the MRSA/SA ELITe MGB assay. It is an automated platform that performs real-time PCR multiplex assays, detecting a specific sequence of the *S. aureus* (proprietary) and the genes *mecA/mecC*. The authors used the assay on 113 respiratory samples, such as sputum, tracheal aspirates, broncho-aspirate and BAL. A total of 23 MRSA and 60 MSSA were identified and the MRSA/SA ELITe MGB assay reached a sensitivity of 95.7% and a specificity of 96.7% for MRSA detection, with two false positives and one false negative (confirmed as a mixed culture with CONS) [81].

#### 4.2.4. Unyvero HPN Application

The Unyvero system (the Unyvero P55 or the Unyvero HPN Application) is a rapid molecular method based on multiple PCRs for the detection of 21 typical lower respiratory tract bacteria and 21 resistance genes, including *mecA/C*. Sun and colleagues evaluated the performance of the Unyvero on BAL fluid samples from patients with lower respiratory tract infections. From the 84 samples analyzed, only 2 MRSA and 1 MSSA were identified. The Unyvero system reached a sensitivity of 100% and a specificity of 98.7% for *S. aureus* detection. The Unyvero detected more than one bacterial species in 9/84 samples, compared with 2/84 samples using the reference methods [82].

#### 4.2.5. GenMark Dx ePlex Blood Culture Identification Gram-Positive (BCID-GP)

The GenMark Dx ePlex blood culture identification Gram-positive (BCID-GP) Panel allows the detection of 20 Gram-positive bacterial species and the resistance genes *mecA/C* and *vanA/B* in positive blood cultures. The system is based on multiplex DNA amplification through competitive DNA hybridization and following electrochemical detection. Carrol et al. used this system in 1297 positive blood cultures. Routine methods detected 194 MRSA and 97 MSSA and the sensitivity of the BCID-GP Panel for MRSA detection was 97.9%. The system could detect multiple staphylococcal species in the same blood culture. However, a known limitation of this method (common to other similar systems) is that in the case of multiple staphylococcal species which also have a *mecA*-positive result, it is not possible to assign to which species the *mecA* gene belongs (*S. aureus* or CONS) [83].

#### 4.2.6. GENECUBE (TOYOBO Co., Ltd., Osaka, Japan)

Hida evaluated the performance of the GENECUBE (TOYOBO Co., Ltd., Osaka, Japan) assay, a fully automated system to detect MRSA or MSSA isolates directly from positive blood cultures. The amplified target genes *mecA* and *nuc* (*S. aureus* specific) are hybridized with fluorescent oligonucleotides and are then detected by changes in fluorescence intensity. The authors analyzed 263 blood cultures and identified 44 MRSA, 56 MSSA, 2 mixed cultures and 161 CONS. The sensitivity and specificity of the GENECUBE were 100%. The system seems to be accurate, rapid (52 min) and reliable but does not detect the *mecC* gene [84]. 

Several other commercial molecular tests exist for the detection of resistance in *S. aureus*. For a description of those systems, please refer to these four reports [19,20,44,98].

### 4.3. In-House Molecular Methods

Several in-house methods have been developed to detect MRSA. The advantages of those methods can be the cost reduction for laboratories which cannot buy expensive commercial assays and the increased flexibility in changing protocols or antibiotics. Therefore, those methods might be better suited for small/local laboratories or laboratories with fewer financial resources which do not have to process a large number of clinical samples. However, in-house methods are non-automated and usually require a longer TAT and it is difficult to compare and reproduce results across different laboratories. 

Galia and co-authors developed a triplex real-time PCR (for screening purposes) for the simultaneous differentiation of MRSA, MSSA and *mecA* positive-CONS isolates and the presence of Panton-Valentine leucocidin directly on rectal and pharyngeal samples. The authors tested this PCR in 42 rectal and 38 pharyngeal samples. A total of 13 MRSA, 2 MSSA and 58 *mecA*-positive CONS were identified by both the reference methods and the real-time PCR. The assay is rapid (TAT: 3 h), accurate, and can be easily implemented to process samples that are strongly contaminated without any sample pretreatment [85]. McClure et al. developed an in-house method for the rapid (8–9 h) detection of MRSA directly from clinical samples. The test includes two PCRs: an initial long-range PCR and a second real-time PCR. The PCR targets the *mecA/C* genes and *orfX* integration genes of the SCC*mec* element. When tested in 88 isolates from nasal, throat, axilla, vaginal or wound samples, 23 MRSA were identified and the assay reached a sensitivity of 100% and a specificity of 97.0% for MRSA detection. The assay performed well in different types of clinical samples but has a longer TAT compared to similar methods and is laborious [86]. Shanmugakani developed a PCR-dipstick to detect MRSA (*mecA*) from positive blood cultures. The authors validated this PCR in 48 already known *S. aureus* isolates (including 33 MRSA) from various clinical samples and in two blood cultures spiked with one MRSA and one MSSA, obtaining 100% sensitivity and specificity with a TAT of 3 h [99].

### 4.4. Loop-Mediated Isothermal Amplification (LAMP)-Based Methods

LAMP can also be used to amplify target sequences/genes. In LAMP, six primers are needed, providing a high specificity. During the assay, two types of elongation reactions amplify the target genes at a fixed (isothermal) temperature. The LAMP can produce a high quantity of target DNA, can be visualized with the naked eye and does not need thermocyclers (as in the case of PCR), since all reactions happen at the same temperature [100]. Kashani and colleagues developed a method based on duplex LAMP for *mecA* and *spa* gene detection. They analyzed 53 clinical isolates (37 MRSA and 16 MSSA) and the LAMP reached a sensitivity of 100% and a specificity of 75.0% for *mecA* detection with a TAT of 188 min [101].

The eazyplex ^®^ MRSA is a LAMP-based portable system that can detect *S. aureus*, *Staphylococcus epidermidis* and the *mecA/C* genes directly in nasal samples or positive blood cultures. Leikem et al. conducted a retrospective observational study to evaluate the performance of eazyplex ^®^ MRSA. From the 797 blood cultures analyzed, 32 MRSA and 199 MSSA were identified through standard methods. The sensitivity and specificity of the eazyplex ^®^ MRSA for MRSA detection were 100% and 99.7%, respectively. In addition, in 190 patients, the authors evaluated the clinical impact of using the eazyplex ^®^ MRSA compared to routine methods. In patients managed according to the results of the eazyplex ^®^ MRSA, the TAT was around 17 h shorter, decreasing from 41.5 to 24 h and the time to the initiation of appropriate treatment was also shorter [87]. The assay provided excellent performance for MRSA detection, but could be optimized for CONS staphylococci, since 16 false positives (out of 566 CONS) were detected, possibly due to *mecA* polymorphisms in CONS (frequently observed [102]), unrecognized polymicrobial infections (commonly observed in BSI [103,104]) or CONS contaminations during sample collection procedures [105].

### 4.5. Whole-Genome Sequencing (WGS)

The routine implementation of WGS is carried out in a few settings and mostly for outbreak investigation or molecular surveillance [106]. WGS is the method with the highest resolution, since it can detect single nucleotide polymorphisms (SNP)s and it can detect all known resistance-associated genes and their variants. Other advantages of using WGS are as follows: WGS data can be stored and re-analyzed if new resistance mechanisms are discovered, or to predict the efficacy of new antibiotics; WGS data can be shared—as opposed to isolates—through laboratories, reducing costs and shipping-related hazards; and comparing WGS data, the genetic relatedness between isolates can be determined in outbreak investigations. However, the routine implementation of WGS in clinical practice has the following barriers: the large amount of data that need to be analyzed and stored, high costs, long TAT, the required bioinformatics experience, the complex workflow, screening for contaminants and resistance genes located in plasmids which might result in low sequencing coverage and be excluded from the analysis. As for other molecular methods, the resistance genes have to be known; uncharacterized or new resistance genes are difficult to detect. Lastly, changes in gene expression cannot be detected [107]. 

Several platforms exist for WGS; Illumina is the most common, is cheaper, provides short sequencing reads (ca. 200 bp) and has a higher accuracy. However, reads are too short to distinguish plasmid from chromosomal DNA and the TAT is 24 h. PacBio and Nanopore produce longer reads (5–10 kb) and are usually more expensive but have a shorter TAT compared to Illumina (PacBio, 0.5–10 h; Nanopore, real-time results). Today, several tools/pipelines (reviewed here [108]) exist to extract resistance-associated genes from the WGS data of different pathogens, including *S. aureus*. Several studies highlighted the general good performance of the WGS-based prediction of antibiotic resistance. Bortolaia et al. developed ResFinder, an online database of antimicrobial resistance genes and mutations in clinically relevant microorganisms. In ResFinder, it is possible to identify specific antimicrobial resistance genes by uploading WGS data. The overall genotype–phenotypic correlation (gene/mutation versus phenotypic AST) was 96.0%, ranging from 76.2% for tetracycline to 100% for cefoxitin and vancomycin [27]. In another study, molecular WGS-based resistance was compared with phenotypic AST, resulting in a sensitivity and specificity of 98.5% and 99.9%, respectively [109]. A similar molecular–phenotypic resistance correlation was observed by Cunningham [110]. 

Brown et al. prospectively performed WGS (and standard methods) on all MRSA isolates in two weeks. They used the Next Gen Diagnostics bioinformatic tool to automatically analyze sequencing data and to confirm the *S. aureus* species, detect the *mecA/C* genes, determine isolates’ relatedness and predict antibiotic resistance. The platform performed all those analyses automatically in 30 s (per sample). In terms of antibiotic resistance, full concordance was observed between the bioinformatic tool and the standard method. Both methods detected two MRSA clusters of three patients. The study showed that one of the impediments to the routine implementation of WGS (the bioinformatics expertise required for data analysis) could be overcome by such an automatic tool [111].

Normally, WGS is performed on isolated bacterial cultures. Those pure bacterial cultures are usually a subculture from the primary clinical culture (see Section 3.1). To reduce the TAT, Blane et al. applied WGS directly on *S. aureus* colonies grown on the primary clinical culture. One challenge of applying this method is that those primary clinical cultures contain colonizers or contaminants and might not have a clear isolated colony to be selected for WGS. The authors tried this “colony pick sequencing” in 30 MRSA, and satisfactory results (in terms of coverage, read quality, presence of contaminants) were obtained for MRSA confirmation [112]. By modifying the Illumina protocol, in another study the authors were able to reduce the TAT for MRSA isolate sequencing (*n* = 25) to 24 h from DNA extraction to WGS results [113].

WGS can also be conducted directly on clinical samples in the so-called metagenomics sequencing; this can reduce the TAT since it is culture-independent but has more DNA from contaminants, other bacteria and human DNA and therefore, it requires a cleaning process to distinguish bacterial DNA from human DNA. In addition, what to do if reads from multiple relevant pathogens are identified through metagenomics sequencing remains a clinical challenge. Platforms exist that can automatize the process of metagenomics sequencing such as IDbyDNA, CosmosID, One Codex, and Karius [107]. 

## 5. Emerging Methods for Antimicrobial Resistance Detection in *S. aureus*

In this section is included studies reporting the following:

- Emerging technologies for the detection of antibiotic resistance in *S. aureus*, largely focused on MRSA;
- Methods at the proof-of-principle stage, and/or those that have not yet been tested in a large collection of isolates;
- Methods allowing AST outside of the microbiological laboratories, i.e., point-of-care (POC) systems.

Some of those POC systems are microfluidic-based. Microfluidics implies a small system where a small amount of sample can be handled in miniaturized fluidic channels, permitting AST. Advantages of having POC microfluidic devices (also referred to as lab-on-a-chip) are as follows: easy to use even by non-trained personnel, rapidity, high throughput analysis, low cost, automation, multiplexing, and portability [18]. Challenges of implementing microfluidic POC systems in routine diagnostics are as follows: the need for specific material with certain features (e.g., electrical conductivity), realistic portability, and the rise of new resistance genes/mechanisms that will impede permanency (and affect the median life) of those devices [26,114]. Khan gave a review focused on the different types of microfluidic systems for AST [114]. An overview of the emerging methods (and their performance) used by the studies selected for this narrative review is given in Table 4. 

### 5.1. Spectroscopy-Based Methods

Spectroscopy is the study of the interaction between radiation and matter. Raman spectroscopy is one spectroscopy technique based on the inelastic scattering of light that provides a spectral fingerprint to identify/differentiate molecules, but also different bacteria and phenotypes (e.g., resistant or susceptible). As different bacterial phenotypes are characterized by different molecular compositions (in terms of nucleic acids, proteins, etc.), those differences will be reflected in different Raman spectra. Despite progress in identifying bacterial spectral fingerprints, more representative and comprehensive databases are needed, along with mathematical models to interpret data [117,129]. Surface-enhanced Raman spectroscopy (SERS) is a type of Raman spectroscopy technique where Raman scattering is enhanced and target/bacteria are identified by their vibrational fingerprint when bacteria are immobilized on nanostructured metallic surfaces. The SERS spectra consist of vibrational bands [118]. 

Potluri et al. combined a duplex PCR (*mecA* and *femA* genes) and SERS nanotags. First, PCR is performed and target genes are amplified. Then, the PCR products are inoculated with gold nanotags, which function as detection probes and hybridized the PCR product. Streptavidin-magnetic beads are then added to the solution and react with the PCR product–nanotags complex, producing a signal detected by the Raman microscope. The author tested this system in 14 MRSA isolated from various clinical samples and four non-staphylococcal species and the system correctly discriminated MRSA from non-MRSA isolates [115]. Li et al. differentiated MRSA from MSSA isolates in blood samples by magnetic separation and the SERS method. First, polyethyleneimine-modified magnetic microspheres (Fe3O4@PEI) were used to capture bacteria directly on blood samples. After 15 min of incaution with Fe3O4@PEI, the complex Fe3O4@PEI-*S. aureus* (magnetically isolated bacteria) were plated in agar with and without antibiotics and incubated overnight. Then, using SERS fingerprints from a single colony, 11 MSSA and 13 MRSA could be correctly identified by analyzing differences in Raman peaks regarding amino acids, lipids and nucleic acid content [116].

Ho et al. developed a database of Raman spectra for 30 clinically relevant bacteria, (including MSSA and MRSA) and applied deep learning techniques to analyze Raman spectra to identify bacteria and detect antibiotic resistance. The authors tested this dataset of Raman spectra directly in five MRSA and five MSSA isolated from various clinical samples (blood cultures, sputum, wound). The method detected MRSA with an accuracy of 65.4% [117]. In another study, the authors observed that if *S. aureus* is treated with antibiotics, the intensity of specific SERS spectra declined after two hours and this information can be used to discriminate MRSA from MSSA [130]. Han and colleagues used this approach to detect MRSA and MSSA isolates in positive blood cultures within 4 h from the positivity signal. Out of 57 positive blood cultures, 15 MRSA and 17 MSSA were identified through standard methods and the SERS protocol had a sensitivity of 93.3% (14/15) and a specificity of 94.1% for MRSA detection [118].

Kochan and coauthors used infrared and Raman spectroscopies combined with chemometric analysis to discriminate MRSA/MSSA by analyzing the changes in chemical composition in one MSSA and one MRSA that were exposed and non-exposed to oxacillin. In the MSSA isolate, the authors identified decreased nucleic acid content, changes in protein composition (α-helix/β-sheet ratio) and changes in carbohydrate composition, suggesting oxacillin activity (i.e., susceptibility). In contrast, no changes were observed between the MRSA isolate exposed or non-exposed to oxacillin [131]. Ciloglu et al. used SERS combined with several machine learning approaches to identify MSSA and MRSA isolates. After overnight culture, *S. aureus* isolates were incubated with Ag nanoparticles and then subjected to SERS measurement. A total of ten MRSA, three MSSA and four negative controls were analyzed and 230 spectra were acquired from those isolates. These spectra could discriminate MRSA from MSSA with an accuracy of 97.8% using the k-nearest neighbors machine learning approach [119]. From the same group, an alternative approach was carried out using SERS combined with another deep learning technique (deep neural network). A total of 19 MRSA from 3 MSSA were analyzed and 33.975 SERS spectra were acquired from those isolates. Those spectra could discriminate MRSA from MSSA with 97.7% accuracy [132].

Regarding the resistance to vancomycin, with routine and automated microbiological tests, it is not possible to distinguish vancomycin-susceptible *S. aureus* (VSSA) isolates from the heterogeneous hVISA. Wongthong et al. used the attenuated total reflection–Fourier transform infrared (ATR-FTIR) spectroscopy to differentiate VSSA from hVISA. A suspension of an isolated *S. aureus* was spotted on the ATR-FTIR spectrometer. From 59 clinical MRSA (31 VSSA and 28 hVISA), 531 spectra were acquired and analyzed. Partial least square discriminant analysis was performed on a subset of ten VISA and ten hVISA and could detect differences in spectra that could differentiate all isolates. For example, hVISA isolates had an increase in the cell wall band (peptidoglycan content in the cell) compared to VSSA, which was coherent with the cell wall thickening of hVISA isolates [120].

### 5.2. Biosensor-Based Methods, Microfluidic-Based Methods

In a biosensor, a bioreceptor (e.g., a DNA probe of 15–50 nucleotides, an antibody, or an enzyme) is immobilized on the surface of the sensor. If the analyte (e.g., *mecA* gene) binds to the bioreceptor, a transducer converts this interaction into a signal that can be displayed and quantified. Biosensors are usually nanomaterial-based [133]. Biosensors can be phenotypic (if they detect phenotypical features such as growth in the presence of antibiotics or cell wall components) or genotypic (if they detect genes) [134]. In recent years, biosensor-based systems showed promising results for the rapid, portable, easy and low-cost detection of pathogens, making them good candidates for POC diagnostics. The challenges of developing biosensors are as follows: a DNA amplification step is often required to enhance the signal intensity and detect low concentrations of analytes (bacteria or genes) in clinical samples; and currently, multiplexing approaches in biosensors are less common compared to PCR assays [81,92,133]. In 2019, Gill and colleagues reviewed the principal optical and electrochemical sensor technologies based on nanomaterials to detect MRSA [133]. In 2021, Reynoso and colleagues provided a review of AST using chemosensors and biosensors [134]. Studies not included in those two reviews, along with newer studies focused on *S. aureus*, are included in this narrative review.

Ozkaya and colleagues developed surface plasmon resonance phenotypic biosensors, which detect changes in the cell wall composition between MSSA and MRSA exposed to cefoxitin, an antibiotic affecting the synthesis of the cell wall. Cefoxitin exposure modifies the cell wall composition and therefore, MRSA can be differentiated from MSSA based on different refraction indexes detected by the sensor. The biosensor correctly identified ten MRSA and ten MSSA clinical isolates compared to the reference method. The system could be adapted to detect multiple pathogens [121]. Another example of a phenotypic biosensor is the fluorescence resonance energy transfer (FRET) probe-based AST method. The method is based on the principle that living *S. aureus* secretes micrococcal nucleases (a known *S. aureus* biomarker) in the extracellular environment. A bacterial suspension from an isolated culture is inoculated in microplates containing the antibiotic being tested and the specific *S. aureus* FRET probe. *S. aureus* surviving in the presence of antibiotics secretes the micrococcal nucleases that cleave the FRET probe, releasing a fluorescence signal. This fluorescent signal is measured and the respective antibiotic MIC is determined in 4–6 h. This FRET probe reached 100% sensitivity and specificity when tested on ten clinical MRSA and MSSA isolates [122].

Nemr and colleagues developed a microfluidic nanoparticle-based device for MRSA screening directly from nasal samples, with minimal sample processing. MRSA samples are incubated in the microfluidic device and are captured by magnetic nanoparticles bound with PBP2a antibodies conjugated with alkaline phosphatase. The authors tested the system in 30 nasal samples and it correctly identified 11 MRSA and 19 non-MRSA isolates [123]. Patel and colleagues developed an electrochemical biosensor to detect one MRSA strain using the bacteriophage SATA-8505 [135]. Maldonado and co-authors developed a nanophotonic biosensor which uses a bimodal waveguide interferometer to differentiate MRSA from MSSA, based on PBP2a protein detection [136]. A last example of a phenotypic biosensor, based on a lateral flow immunoassay that detects the PBP2a, was developed by Amini and coauthors [137].

Mohamed et al. developed a colorimetric nanodiagnostic system for the identification of seven bacterial species and resistance genes (*mecA*, *blaZ*, *vanA*, *vanB*, *tetK*, *tetM*, *ermA*, and *ermC*). The system is based on a multicomponent nucleic acid enzyme−gold nanoparticle (MNAzyme-GNP) and can be used directly on clinical samples. DNA is extracted and then amplified through isothermal amplification. The amplified DNA target is detected by the MNAzyme-GNP platform based on colorimetric change. The authors tested the system in 50 clinical samples (nasal, groin, axilla, and wound swabs) for *mecA* detection. Thirty samples were *mecA*-positive and 20 were *mecA*-negative and the system reached 90% sensitivity and 95% specificity compared to *mecA*-PCR [124].

Chen et al. combined the LAMP technique with the detection of LAMP target genes *mecA* and *femA* through a nanoparticle-based lateral flow biosensor. The author tested this method in 63 clinical whole blood samples from patients suspected of being infected with *S. aureus*. The biosensor correctly detected 12 MRSA, 16 MSSA and 35 *S. aureus*-negative isolates, reaching a sensitivity and specificity of 100% compared to cultures [125]. Several other molecular biosensors have been described, all targeting the *mecA* gene: a nanoelectrokinetic sensor [138], a capacitive biosensor tested on a saliva sample [139], a portable and smartphone-controlled colorimetric LAMP device [140], a hairpin probe-mediated DNA circuit based on exonuclease III and DNAzyme tested on *mecA*-spiked serum samples [141], a colorimetric paper-based analytical device [142], a ligation chain reaction electrochemical sensor tested on joint fluid samples [143] and a microfluidic biochip based on roll-to-roll UV nanoimprint technology tested on nasal, throat and inguinal samples [144].

### 5.3. Other POC Systems

Meng and colleagues developed a microfluidic LAMP-based device capable of detecting *S. aureus*, *S. epidermidis*, *Staphylococcus haemolyticus*, and *Staphylococcus hominis* (by targeting the variable region of the *femA* gene) and the *mecA* gene. The other staphylococcal species are clinically relevant for BSI. The authors tested this assay in 102 cerebrospinal fluid (CSF) positive cultures. After a CSF culture was flagged as positive, an aliquot of this suspension was used in the microfluidic device. The assay correctly identified all species, 4 MRSA and 11 MSSA, in 70 min. In addition, the system also detected six mixed cultures (*S. epidermidis*–*S. hominis*) [126]. Neil et al. proposed a method for the detection of PBP2a and PBP2c proteins through intact protein liquid chromatography and tandem mass spectrometry. They evaluated the method in 25 representative MRSA isolates containing different SCC*mec* types, 3 MSSA isolates and 13 clinical isolates (12 MRSA and 1 MSSA). All those isolates were correctly identified compared to reference methods [127].

Brown and co-authors developed a bacteriophage-based diagnostic test to screen for MRSA nasal colonization. Two NanoLuc luciferase reporter phages were used, ISP and MP115. Overnight, *S. aureus* cultures were transferred on 96-well strips and then mixed with the phage cocktail. The final results were available in 6 h. In 40 nasal samples (all MSSA), the test detected 4 false-positive MRSA (specificity 90%). When the authors spiked the 40 nasal swabs with MRSA, the test detected all 40 MRSA (sensitivity 100%) [128]. Choopara et al. developed a fluorometric cellulose paper-based LAMP system for *mecA* detection in positive blood cultures [145]. Hilton et al. used dielectrophoresis to identify possible changes (to be used as a marker or for developing rapid AST tests) in biophysical properties between one MSSA and one MRSA [146]. Schulz and coauthors developed a POC system that can detect MRSA, MSSA and *mecA*-positive CONS in single cells directly from nasal samples [147].

## 6. Conclusions

In this narrative review, I reviewed some of the recent phenotypic and molecular methods to detect antimicrobial resistance in *S. aureus*. As expected, the large majority of the analyzed studies focused on MRSA detection. MRSA impact on morbidity and mortality still remain high, especially in hospital settings [148,149,150,151]; the rapid and accurate diagnosis of MRSA makes sure that patients receive the proper antibiotics and improves patient outcomes [16,111]. In addition, the surveillance of antibiotic resistance in *S. aureus* remains crucial due to its historical capacity to rapidly adapt to new antibiotics (see introduction).

A wide range of methods and emerging technologies exist to detect resistance in *S. aureus* isolates. In many studies included in this narrative review, the identification and resistance determination of *S. aureus* were performed with at least two methods (see for example [78,81]). A single method that is able to both identify and perform AST would greatly simplify diagnostic workflows, especially if it could detect several pathogens. In this view, the Accelerate PhenoTest or the MALDI-TOF, if optimized for AST, can have a large impact on routine diagnostics. Another option would be to use a solid gold-standard method and make it automated, to reduce hands-on time, to improve inter-laboratory reproducibility and to standardize the interpretation of results. The Copan WASP, an automated disk diffusion method, fulfills those requirements. The rapid BMD method and the rapid EUCAST disk diffusion combine the solidity of gold-standard methods and reduce the TAT; those two options are cheaper than automated AST systems and therefore are ideal in low-resource settings or small/local laboratories with a low diagnostic workload. 

Molecular methods greatly reduced the TAT for MRSA identification. Several studies highlighted the clinical benefits of using the GeneXpert system compared to routine methods [76,78,79,80]. A single molecular method able to detect several pathogens would be an added value compared to the GenXpert; for example, the GenMark Dx ePlex [83]. One problem in *S. aureus* is the frequent contamination with other CONS in clinical samples; if a molecular method detects the *mecA* gene in a mixed sample/culture, we cannot determine whether the *mecA* belongs to *S. aureus* or CONS, since the *mecA* gene (and its variants) are also present in CONS [18]. In those mixed cultures, a confirmatory (phenotypic) test might be needed to resolve the problem. Another issue complicating diagnostic tests is the presence of *mecA* variants [58,74,75] and the emergence of new SCC*mec* elements [19,73]. The routine implementation of WGS in routine diagnostics for *S. aureus* resistance detection is complicated, especially because so far there is a lack of evidence showing the clinical benefits of implementing WGS in terms of reduced TAT, reduced costs or better resistance determination compared to other methods. The Nanopore MinION platform looks promising from a diagnostic perspective due to its small size, portability, rapid TAT and real-time delivery of results [107]. Tools permitting the automatic identification of resistance genes by uploading WGS data reduce the bioinformatics expertise required to analyze WGS data to predict antibiotic resistance. Those tools can be expanded and updated, as long as more information about resistance is discovered [27,108]. Metagenomic sequencing in clinical samples might represent the near future of implementing sequencing technologies in routine diagnostics.

Compared to previous reviews focusing on MRSA diagnostics [18,19,20], in this narrative review I found many studies reporting molecular methods moving towards POC diagnostics (but I recognize that this could be due to differences in a searching strategy or in the subjective selection of studies to report). However, all those studies are still at the proof-of-principle stage and need to be tested in a variety of isolates. Those methods are based on biosensors in microfluidic systems and could reduce the TAT even more. The capacity of a biosensor-based POC system to detect several pathogens might be an added value since many of those emerging technologies detect only one pathogen (e.g., *S. aureus*) and one antibiotic resistance (e.g., *mecA* gene). The MRSA POC might be particularly useful for admission screening or screening before surgery [18].

This narrative review has three major limitations. The first limitation is the typical subjectivity of narrative reviews in terms of study selection and the extent to which each study is discussed. The search strategy and the study selection have not been double-checked by another author and the process is less standardized compared to systematic reviews [152]. Second, it is difficult, if not impossible, to have a homogeneous report due to the large variety of available methods. For example, the sensitivity, and specificity of the rapid BMD method [30], are conceptually different from the sensitivity and specificity of Raman spectroscopy and deep learning approaches [118]. Another example is when studies that assess diagnostic performance differ in their definition of TAT; some studies consider only the duration of the test, while others consider test duration plus hands-on time and sample preparation (see for example [76,77,79,80]). Additionally, in studies focusing on biosensors or lab-on-a-chip devices in particular, it is sometimes hard to extract accurate microbiological information from the study, such as criteria for isolate selection, the number and type of isolates, whether the test is performed on an isolated culture or a clinical sample, or if any sample pretreatment is required, etc. This might be due to the backgrounds of the authors reporting the research, since they might focus more on technicalities than on providing full microbiological details. In addition, although standards exist for the reporting of diagnostics studies [153], as observed by Poole et al. [154], I also found that not all studies adhere to those standards, and they differ in reporting sensitivity, specificity, CA, VME or ME. Third, few studies reported the full range of the clinically important criteria of the specific method, such as sensitivity, specificity, TAT improvement (or not), time to targeted therapy, eventual reduction in hospitalization or stays in the ICU, eventual reduction of therapy duration with broad-spectrum antibiotics, or details of saved costs for the patient/healthcare system. Therefore, there is generally a lack of prospective assessment of the implications of the specific method on clinical outcomes. I see that this would be an ideal solution and that there might be several practical/logistics reasons for not being able to report all details; however, having a more heterogeneous pool of studies might allow for a more realistic comparison and a better judgment of whether a method can be implemented in routine diagnostics. 

The large variety of available methods for antimicrobial resistance detection in *S. aureus* is probably going to remain as such, and may even increase. The different clinical settings where a method can be implemented needs to be considered: low vs. high resources, high-workload vs. low-workload, and centralized laboratories testing the full range of pathogens/antibiotics vs. POC systems that are closer to patients that are focused only on one pathogen and one resistance. Ideally, the best method for detecting resistance in *S. aureus* should have the following features: the ability to detect resistance directly on the clinical sample, being automated, portability, requiring minimal resources, easy to use even by non-trained professionals, rapid TAT and flexibility in changing antibiotics to test. Even if at the moment there is no test fulfilling all those criteria, this should serve as a roadmap to follow [26,114,155]. Designing and improving diagnostic methods for antimicrobial resistance detection in *S. aureus* is one of the multiple ways to reduce the burden of antibiotic resistance.

## Figures and Tables

**Table 1:** Overview of the phenotypic methods for antimicrobial resistance detection in *Staphylococcus aureus* used by the studies selected for this narrative review. A summary of the major diagnostic performance on *S. aureus* isolates is shown, as compared with the reference gold-standard method (if performed) of each specific study.

Phenotypic Method Used	Principle of the Method	On Culture/on Clinical Sample	TAT ^1^	Brief Advantages/Disadvantages +/−	No. and Type of *S. aureus* Anlyzed ^2^	Major Diagnostic Performance ^3^	Reference
Chromogenic media	Selective media, colorimetric colony detection	Nasal swabs, positive blood culture	24 h	+ Easy to perform and interpret, low-cost − No MSSA identification	72 MRSA 28 MSSA	Sensitivity 100% Specificity 78.6%	[28]
					19 MRSA 29 MSSA	Sensitivity 78.9% Specificity 41.3%	[29]
Rapid BMD	Colorimetric assay, MTT dye as oxidation-reactions indicator	Culture	7 h	+ Easy to perform, rapid, low-cost − Larger validations needed	21 MRSA 19 MSSA	Sensitivity 100% Specificity 94.7%	[30]
Disk diffusion on Mueller-Hinton rapid agar MHR-SIR	Disk diffusion with shorter incubation	Positive blood culture	6–8 h	+ Easy to perform, rapid, low-cost − Larger validations needed	23 *S. aureus* tested for several antibiotics	CA 97.8% mEs 1.9% VME 0.3%	[31]
					29 MRSA 50 MSSA	TAT 17h shorter, better therapy de-escalation	[32]
Rapid AST	Disk diffusion with shorter incubation	Positive blood culture	4–6 h	+ Easy to perform, rapid, low-cost − Low growth, diameters margins less demarcated	24 MRSA 313 MSSA	Sensitivity 100% Specificity 100%	[33]
					14 MRSA 183 MSSA	Sensitivity 100% Specificity 99.4% ATU 0.5%	[34]
					9 MRSA 212 MSSA	Sensitivity 100% Specificity 99.1% ATU 0.4% ME 0.5%	[35]
VITEK 2	Turbidimetric monitoring of bacterial growth	Culture	4–11 h	+ Automated, rapid, standardized interpretation − Fixed range of testable antibiotics, less accurate for linezolid/vancomycin	72 MRSA 28 MSSA	Sensitivity 97.2% Specificity 100%	[28]
					27 MRSA resistant to linezolid	MEs 85.2%	[36]
					28 MRSA with a vancomycin MIC ≥ 2	MEs 46.4%	[37]
BD Phoenix	Turbidimetric and colorimetric growth detection	Culture	6–16 h	+ Automated, rapid, standardized interpretation − Less accurate for SXT	642 *S. aureus* analyzed for SXT resistance	CA 91.9% MEs 7.9% mEs 0.3%	[38]
Copan WASP Colibri	Automated disk diffusion	Culture	16 h	+ Automated, flexible, detect heteroresistance and mixed cultures − Should be compared with standard disk diffusion	107 *S. aureus* tested for several antibiotics	CA 99.9%	[39]
Accelerate PhenoTest™ BC	Single-cell analysis, fluorescent in situ hybridization	Positive blood culture	7 h	+ Automated, monomicrobial call, rapid, identification and AST in the same platform	98 MRSA 86 MSSA	Sensitivity 100% Specificity 98.8% CA 99.5%	[17]
					22 MRSA 2 MSSA	Sensitivity 100% Specificity 100%	[40]
PBP2a latex agglutination assay	Particles with monoclonal antibodies, agglutination reaction	Culture	5 m	+ Rapid, easy to perform and interpret − no PBP2c detection	95 MRSA 10 MSSA	Sensitivity 98.95% Specificity 77.8%	[41]
PBP2a SA culture colony test	Monoclonal antibodies immobilized in a membrane	Culture	5 m	+ Rapid, easy to perform and interpret − no PBP2c detection	63 *mecA*-MRSA 10 *mecC*-MRSA	Sensitivity 100% Specificity 100% for *mecA*-MRSA	[42]
	On shortly incubated cultures	4–6 h Culture	5–10 m	+ More rapid than standard protocol − no PBP2c detection	25 MRSA 13 MSSA	Sensitivity 96.0% Specificity 92.0%	[43]

^1^ Time to AST results, not considering the time needed to obtain an isolated culture, if needed. ^2^ Identified as *S. aureus* by the reference method used in the respective study. ^3^ Calculated for MRSA detection, unless other antibiotics tested. TAT: turnaround time; h: hours; m: minutes; MRSA: methicillin-resistant *S. aureus*; MSSA: methicillin-susceptible *S. aureus*; BMD: broth microdilution method; CA: categorical agreement; VME: very major error; ME: major error; mE: minor error; AST: antimicrobial susceptibility testing; ATU: area of technical uncertainty; SXT: Trimethoprim/Sulfamethoxazole; PBP: penicillin-binding protein.

**Table 2:** Overview of MALDI-TOF MS approaches for antimicrobial resistance detection in *Staphylococcus aureus* used by the studies selected for this narrative review. A summary of the major diagnostic performance on *S. aureus* isolates is shown, as compared to the reference gold-standard method used in each specific study.

MALDI-TOF System Used	Principle of the Method	On Culture/on Clinical Sample	TAT ^1^	Brief Advantages/Disadvantages +/−	No. and Type of *S. aureus* Analyzed ^2^	Major Diagnostic Performance ^3^	Reference
MALDI Biotyper ^®^ CA System (Bruker Daltonics)	21 peaks to discriminate MRSA/MSSA	Culture	Few m	+ Use a combination of peaks − Low specificity	181 *S. aureus*	Sensitivity 87.6% Specificity 71.4%	[64]
MALDI Biotyper ^®^ CA System (Bruker Daltonics)	Peptide PSM-*mec* and δ-toxin peaks for MRSA identification	Culture	Few m	+ Sensitive for SCC*mec* type II, III and VIII − Not adapted for other SCC*mec* types	35 MRSA	Sensitivity 40%	[65]
MALDI Biotyper ^®^ CA System (Bruker Daltonics)	Peptide PSM-*mec* peak for MRSA identification	Culture	Few m	+ Good specificity − SCC*mec* types of isolates unknown	241 MRSA 106 MSSA	Sensitivity 60.2% Specificity 100%	[66]
MALDI Biotyper ^®^ CA System (Bruker Daltonics)	m/z 4594 peak for MRSA identification	Culture	Few m	+ Potential MRSA biomarker peak − To be tested in an independent set of data	36 MRSA 31 MSSA	Sensitivity 83.3% Specificity 96.8%	[67]
VITEK MS (BioMérieux)	support vector machine algorithm to identify 38 peaks to discriminate MRSA/MSSA	Culture	Few m	+ Use a combination of peaks − MRSA/MSSA classification model needs optimization	194 MRSA 258 MSSA	Sensitivity 84.0% Specificity 88.0%	[68]
MALDI Biotyper ^®^ CA System (Bruker Daltonics)	DOT-MGA	Positive blood cultures	6 h	+ Rapid; tested with cefoxitin − Could be optimized for additional antibiotics	30 MRSA 14 MSSA	Sensitivity 100% Specificity 100%	[69]
VITEK MS (BioMérieux)	DOT-MGA	Culture	5 h	+ Tested with oxacillin − Could be optimized for additional antibiotics	20 MRSA 20 MSSA	Sensitivity 100% Specificity 100%	[70]

^1^ Time to AST results, not considering the time needed to obtain an isolated culture, if needed. ^2^ Identified as *S. aureus* by the reference method used in the respective study. ^3^ Calculated for MRSA detection, unless other antibiotics tested. MALDI-TOF MS: Matrix-assisted laser desorption/ionization time-of-flight mass spectrometry; DOT-MGA: direct-on-target microdroplet growth assay.

**Table 3:** Overview of the molecular methods for antimicrobial resistance detection in *Staphylococcus aureus* used by the studies selected for this narrative review. A summary of the major diagnostic performance on *S. aureus* isolates is shown, as compared with the reference gold-standard method (if performed) of each specific study.

Molecular Method Used	Principle of the Method	On Culture/on Clinical Sample	TAT ^1^	Brief Advantages/Disadvantages +/−	No. and Type of *S. aureus* Analyzed ^2^	Major Diagnostic Performance ^3^	Reference
Xpert ^®^ SA Nasal Complete (Cepheid)	Real-time PCR for *mecA/C*, *spa* and SCC*mec*-*orfX*	Nasal samples	3 h	+ Clinical outcomes analyzed − 56 invalid results not further analyzed	10 MRSA in 605 nasal samples	Sensitivity 100% Specificity 98.8% PPV 58.8% NPV 100% TAT 41 h shorter	[76]
			85 m	− Unusual reference method	27 MRSA in 500 nasal samples	Sensitivity 51.8% Specificity 100%	[77]
Xpert ^®^ MRSA/SA BC Assay (Cepheid)	Real-time PCR for *mecA/C*, *spa* and SCC*mec*-*orfX*	Positive blood cultures	1.7 h	+ Clinical outcomes analyzed − More resistant isolates needed	1 MRSA 38 MSSA in 264 blood cultures	Sensitivity 100% specificity 100% TAT 24 h shorter, earlier changes in patient management	[78]
			n.a.	+ Clinical outcomes analyzed − Sensitivity/specificity not calculated	37 MRSA 64 MSSA	Time to optimal therapy 20 h shorter, duration of vancomycin therapy 18 h shorter	[79]
Xpert ^®^ MRSA/SA SSTI (Cepheid)	Real-time PCR for *mecA/C*, *spa* and SCC*mec*-*orfX*	BAL samples	68 m	+ Clinical outcomes analyzed − Method not validated in BAL samples	23 MRSA 25 MSSA in 247 BAL samples	Sensitivity 95.7% specificity 98.2% Time of linezolid/vancomycin treatment 40 h shorter	[80]
Hologic Panther Fusion ^®^ MRSA	PCR and Invader chemistries for *mecA/C*, *gap* and SCC*mec*-*orfX*	Nasal samples	<3 h	+ Can analyze 350 samples in 8 h − Need comparison with a similar method	30 MRSA 112 MSSA in 434 nasal swabs	Sensitivity 86.7%, specificity 98.8%, CA 97.9%	[72]
MRSA/SA ELITe MGB assay (ELITechGroup)	Real time PCR for *mecA/C* and a *S. aureus* specific sequence	Sputum, tracheal aspirate, BAL	<3 h	+ Accurate − Do not target SCC*mec*-*orfX* junction	23 MRSA 60 MSSA in 113 respiratory samples	Sensitivity 95.7% specificity 96.7% PPV 91.7% NPV 98.3%	[81]
Unyvero HPN Application	Multiple PCRs	BAL fluids	5 h	+ Detect 21 species and 19 resistance genes; mixed cultures detection − More resistant isolates needed	2 MRSA 1 MSSA in 84 BAL fluids	Sensitivity 100% specificity 98.7%	[82]
GenMark Dx ePlex blood culture identification	Hybridization and electrochemical detection, *mecA/C*, *vanA/B*	Positive blood cultures	<2 h	+ Detect 20 Gram-positive species; mixed cultures detection	194 MRSA 97 MSSA in 1297 blood cultures	Sensitivity 97.9% specificity 100%	[83]
GENECUBE (TOYOBO)	*nuc* and *mecA* amplification and, hybridization	Positive blood cultures	52 m	+ Faster than similar methods − No *mecC* detection	44 MRSA 56 MSSA in 263 blood cultures	Sensitivity 100% specificity 100% LOD 12.5 gene copies/test	[84]
Real-time triplex PCR	Targeting *nuc*, *mecA* and *pvl*	Rectal and pharyngeal samples	3 h	+ Applicable in contaminated samples − No *mecC* detection	12 MRSA 3 MSSA in 80 samples	Sensitivity 100% specificity 100% LOD 514 CFU/ml	[85]
Long-range and real-time PCRs	Targeting *mecA/C* and *orfX*	Nasal, wound, axilla, throat samples	8–9 h	+ Perform well in various samples − Slower than similar methods, laborious	23 MRSA in 88 samples	Sensitivity 100% specificity 97.0%	[86]
Eazyplex ^®^ MRSA	LAMP targeting *S. aureus*, *S. epidermidis*, *mecA/C*	Positive blood cultures	1 h	+ Portable; faster than similar methods − Need optimization for CONS	32 MRSA 199 MSSA in 797 blood cultures	Sensitivity 100%, specificity 99.7%, TAT 17 h shorter	[87]

^1^ Time to AST results, not considering the time needed to obtain an isolated culture, if needed. ^2^ Identified as *S. aureus* by the reference method used in the respective study. ^3^ Calculated for MRSA detection, unless other antibiotics tested. PCR: polymerase chain reaction; PPV: positive predictive value; NPV: negative predictive value; n.a.: not available; BAL: bronchoalveolar lavage; LOD: limit of detection; CFU: colony-forming units; LAMP: loop-mediated isothermal amplification; CONS: coagulase-negative staphylococci.

**Table 4:** Overview of emerging methods for antimicrobial resistance detection in *Staphylococcus aureus* used by the studies selected for this narrative review. A summary of the major diagnostic performance on *S. aureus* isolates is shown, as compared with the reference gold-standard method (if performed) of each specific study.

Method Used	Principle of the Method	On Culture/on Clinical Sample	TAT ^2^	Brief Advantages/Disadvantages +/−	No. and Type of *S. aureus* Analyzed ^1^	Major Diagnostic Performance ^3^	Reference
Duplex PCR + SERS	PCR for *mecA* and *femA*, magnetic separation and SERS detection	Culture	1.5 h	+ Customizable − No comparisons with standard methods	14 MRSA	MRSA correctly identified; LOD 10^4^ DNA copies	[115]
SERS based on magnetic microspheres	Magnetic separation with microspheres, SERS fingerprint	Blood samples	24 h	+ No sample pretreatment − No comparisons with standard methods	13 MRSA 11 MSSA in 77 blood cultures	MRSA MSSA differentiated	[116]
Raman spectroscopy + deep learning	Raman spectroscopy and convolutional neural network	Blood cultures, sputum, wound	Few h	+ Label-free − Low accuracy with clinical isolates;	5 MRSA 5 MSSA	Accuracy 65.4%	[117]
SERS	Ag-nanoparticle fixed in nanochannels, spectra measurement	Positive blood cultures	4 h	+ Rapid − Require blood culture pretreatment	15 MRSA 17 MSSA in 75 blood cultures	Sensitivity 93.3%, specificity 94.1%, 1 VME, 1 ME	[118]
SERS + deep learning	SERS and deep neural network	Culture	0.5–1 h	+ Rapid	19 MRSA 3 MSSA	Accuracy 97.7%	[119]
ATR-FTIR spectroscopy	Infrared spectroscopy and multivariate analysis	Culture	15 m	+ Faster than similar methods; few reagents needed − Variability in cell wall thickness to be investigated	10 VSSA 10 hVISA	Sensitivity 100%, specificity 100%,	[120]
Surface plasmon resonance sensor platform	Measure changes in cell wall refractive index	Culture	3 h	+ Easy to use; multiplexing possible; − Bacterial adherence instability	10 MRSA 10 MSSA	Sensitivity 100%, specificity 100%,	[121]
FRET probe-based AST	Micrococcal nuclease detection	Culture	4–6 h	+ Several antibiotics tested	10 MRSA 10 MSSA	Sensitivity 100%, specificity 100%	[122]
Electrochemical biosensor	Magnetic nanoparticles-based detection of PBP2a	Nasal samples	4.5 h	+ Multiplexing possible; minimal samples pretreatment − No MSSA detection (for screening only)	11 MRSA 19 non-MRSA	Sensitivity 100%, specificity 100%, LOD 845 CFU/mL	[123]
MNA-zyme-GNP platform	Amplification of *mecA* and *fib*, gold nanoparticles binding, colorimetric detection	Nasal, groin, axilla, wound swabs	2 h	+ Naked-eye detection; isothermal amplification − Multi-pathogen detection needs validation	30 *mecA*-positive 20 *mecA*-negative	Sensitivity 90%, specificity 95%, LOD 10^2^–10^3^ CFU/mL	[124]
Duplex LAMP + lateral flow biosensor	Targeting *mecA* and *femA*	Blood samples	80 m	+ Naked-eye detection; isothermal amplification; rapid; easy to perform	12 MRSA 16 MSSA in 63 blood samples	Sensitivity 100%, specificity 100% LOD 100 fg of genomic DNA	[125]
Microfluidic device LAMP-based	Targeting *mecA* and the variable region of *femA*	CSF positive cultures	70 m	+ Multiple staphylococcal species detection	4 MRSA 11 MSSA in 102 CSF positive cultures	Sensitivity 100%, specificity 100% LOD 20–200 CFU/reaction	[126]
Tandem mass spectrometry of PBP2a–PBP2c	Liquid chromatography and tandem mass spectrometry	Culture	5–120 m	+ Detection of PBP2c also	37 MRSA + 4 MSSA	Sensitivity 100%, specificity 100%	[127]
Bacteriophage-based MRSA screening	NanoLuc lucipherase reporter phages ISP and MP115	Nasal samples	6 h	+ Low-cost − Slower compared to similar methods	40 spiked MRSA 40 MSSA	Sensitivity 100%, specificity 90% LOD 75–750 CFU per sample	[128]

^1^ Identified by the gold standard methods used in the respective study. ^2^ The time needed to obtain an isolated culture needs to be added. ^3^ Calculated for MRSA detection, unless other antibiotics tested. SERS: Surface-enhanced Raman spectroscopy; ATR-FTIR: attenuated total reflection-Fourier transform infrared; VSSA: vancomycin-susceptible *S. aureus*; hVISA: heterogeneous vancomycin-intermediate *S. aureus*; FRET: fluorescence resonance energy transfer; MNA-zyme-GNP: multi-component nucleic acid enzyme−gold nanoparticle; CSF: cerebrospinal fluid.

## Data Availability

The data presented in this study are available in the main article and in the Supplementary Material S1.

## Supplementary Materials

The following supporting information can be downloaded at: https://www.mdpi.com/article/10.3390/diagnostics12010208/s1, Supplementary Material S1: List of the 126 studies included in the narrative review, with their relative doi.
