# Supplementary material for: Recent Developments in Phenotypic and Molecular Diagnostic Methods for Antimicrobial Resistance Detection in Staphylococcus aureus: A Narrative Review
Source: Diagnostics (Basel). 2022 Jan 15;12(1):208. doi: 10.3390/diagnostics12010208 (PMC8774325; doi:10.3390/diagnostics12010208)
Supplement: Supplementary file 1 [file diagnostics-12-00208-s001.zip › diagnostics-1556843-supplementary.pdf]

Supplementary Material S1. List of the 126 studies included in the narrative review, with their relative doi.

Total studies included from the databases (n=99)

1. Kaprou, 2021 https://doi.org/10.3390/antibiotics10020209
2. Bortolaia, 2020 https://doi.org/10.1093/jac/dkaa345
3. Palavecino, 2020 https://doi.org/10.1007/978-1-4939-9849-4_2
4. Madhavan, 2021 http://dx.doi.org/10.18502/ijm.v13i1.5489
5. Kaya, 2019 https://doi.org/10.18016/ksutarimdoga.vi.457495
6. Mahmoud, 2019 https://doi.org/10.1111/jam.14347
7. Pilmis, 2019 https://doi.org/10.1093/jac/dkz271
8. Åkerlund, 2020 https://doi.org/10.1093/jac/dkaa333
9. Jasuja 2020 https://doi.org/10.1007/s10096-020-04146-6
10. Jasuja 2020 https://doi.org/10.1007/s10096-020-03846-3
11. Yoo, 2020 https://doi.org/10.3343/alm.2020.40.1.57
12. Kuo, 2020 https://doi.org/10.7717/peerj.8963
13. Cherkaoui 2021 https://doi.org/10.1128/JCM.00777-21
14. Cherkaoui 2020 https://doi.org/10.1016/j.cmi.2019.11.008
15. Al Rawahi 2019 https://doi.org/10.1128/JCM.00994-19
16. Khawaja, 2020 10.5455/JPMA.290732
17. Dupieux, 2020 https://doi.org/10.1128/JCM.01346-19
18. Kolesnik, 2021 https://doi.org/10.1128/Spectrum.00462-21
19. Becker, 2020 https://doi.org/10.3389/fmicb.2020.568891
20. Rodriguez-Sanchez 2019 http://dx.doi.org/10.2807/1560-7917.ES.2019.24.4.1800193
21. Yoon, 2021 https://doi.org/10.3390/antibiotics10080982
22. Welker, 2019 https://doi.org/10.3389/fmicb.2019.02711
23. Oviano, 2019 https://doi.org/10.1128/CMR.00037-18
24. Kim, 2019 https://doi.org/10.3390/pathogens8040214
25. Paskova, 2020 https://doi.org/10.1007/s12223-020-00799-0
26. Hu, 2019 https://doi.org/10.3389/fmicb.2019.02504
27. Nix, 2020 https://doi.org/10.3389/fmicb.2020.00232
28. Horseman, 2020 https://doi.org/10.1016/j.diagmicrobio.2020.115093
29. Flores-Treviño, 2019 https://doi.org/10.1038/s41598-019-55430-1
30. Liu, 2020 https://doi.org/10.1002/rcm.8972
31. Sanchez-Carrillo, 2019 https://doi.org/10.1007/s10096-019-03595-y
32. Boland, 2019 https://doi.org/10.1186/s12879-019-4623-x
33. Mizusawa, 2020 https://doi.org/10.1080/14787210.2020.1760842
34. Maurin, 2020 https://doi.org/10.1007/s10096-020-03968-8
35. Liu, 2020 https://doi.org/10.1016/j.jmii.2020.07.021
36. Liu, 2021 https://doi.org/10.2147/IDR.S288991
37. Brahama, 2019 https://doi.org/10.1038/s41598-019-52557-z
38. Boonsiri, 2020 https://doi.org/10.1038/s41598-019-52557-z
39. Goering, 2019 https://doi.org/10.1128/AAC.00558-19
40. Kime, 2019 https://doi.org/10.1128/mBio.01755-19
41. Gargis, 2020 https://doi.org/10.1128/JCM.02038-19
42. Dewar, 2019 https://doi.org/10.1016/j.jhin.2019.08.002
43. McHugh, 2020 https://doi.org/10.1099/jmm.0.001171
44. Drwiega, 2019 10.1097/IPC.0000000000000737
45. Titecat, 2021 https://doi.org/10.3389/fmed.2021.553965

46. Paonessa, 2019 https://doi.org/10.1016/j.chest.2019.02.007
47. Coppens, 2019 https://doi.org/10.1186/s13756-018-0460-8
48. Ayebare, 2019 https://doi.org/10.1186/s12866-019-1566-8
49. Boattini, 2020 https://doi.org/10.1016/j.jhin.2019.12.025
50. Sun, 2021 https://doi.org/10.1007/s10096-021-04259-6
51. Carroll, 2020 10.1128/JCM.01730-19
52. Hilda, 2019 10.1371/journal.pone.0219819
53. Buonomini, 2020 10.3390/diagnostics10100830
54. Galia, 2019 10.3934/microbiol.2019.2.138
55. McClure, 2020 https://doi.org/10.3389/fmicb.2020.01295
56. Shanmugakani 2020, https://doi.org/10.1099/jmm.0.001159
57. Kashani, 2020 10.5812/jjm.96682
58. Leikem, 2000 https://doi.org/10.1007/s10096-019-03773-y
59. Babiker, 2019 https://doi.org/10.1016/j.jgar.2019.04.006
60. Cunningham, 2020 https://doi.org/10.1016/j.diagmicrobio.2020.115060
61. Ransom, 2020 https://doi.org/10.1093/clinchem/hvaa172
62. Brown, 2019 https://doi.org/10.1128/JCM.00858-19
63. Blane, 2019 https://doi.org/10.1093/jac/dkz170
64. Raven, 2019 https://doi.org/10.1128/JCM.00180-19
65. Ho, 2019 https://doi.org/10.1038/s41467-019-12898-9
66. Han, 2020 https://doi.org/10.1038/s41598-020-68855-w
67. Potluri, 2020 https://doi.org/10.1039/C9AN01959F
68. Li, 2019 https://doi.org/10.1007/s00604-019-3571-x
69. Kochan, 2020 https://doi.org/10.1021/acs.analchem.0c00474
70. Ciloglu, 2020 https://doi.org/10.1039/D0AN00476F
71. Ciloglu, 2021 https://doi.org/10.1038/s41598-021-97882-4
72. Gill, 2019 https://doi.org/10.1007/s00604-018-3186-7
73. Reynoso, 2021 https://doi.org/10.3390/chemosensors9080232
74. Ozkaya, 2019 https://doi.org/10.3390/diagnostics9040191
75. Patel, 2021 https://iopscience.iop.org/article/10.1149/1945-7111/abef85
76. Mohamed, 2021 https://doi.org/10.1021/acsnano.0c09902
77. Oueslat, 2021 https://doi.org/10.3390/chemosensors9050097
78. Mahadhy, 2020 https://doi.org/10.1016/j.btre.2020.e00568
79. Ma, 2019 https://doi.org/10.1039/C9LC00797K
80. Pan, 2021 https://doi.org/10.1039/D1AN00028D
81. Suea-Ngam, 2021 https://doi.org/10.1002/adhm.202001755
82. Chen, 2021 https://doi.org/10.1039/D1AN00350J
83. Chen, 2020 https://doi.org/10.1186/s13756-020-00774-x
84. Meng, 2020 https://doi.org/10.3389/fmicb.2020.01487
85. Choopara, 2021 https://doi.org/10.1021/acssensors.0c01405
86. Maldonado, 2020 https://doi.org/10.1039/C9AN01485C
87. Amini, 2020 https://doi.org/10.1080/17458080.2020.1775197
88. Hilton, 2020 https://doi.org/10.1039/C9AN01449G
89. Neil, 2021 https://doi.org/10.1038/s41598-021-97844-w
90. Schulz, 2020 https://doi.org/10.1039/D0LC00294A
91. Khan, 2019 https://doi.org/10.1007/s10529-018-02638-2
92. Nemr, 2019 https://doi.org/10.1021/acs.analchem.8b04792
93. Toren, 2020 https://doi.org/10.1039/D0LC00751J
94. Wongthong, 2020 https://doi.org/10.1007/s11274-019-2788-5

95. Wu, 2021 https://doi.org/10.1186/s13756-021-00967-y
96. Ersoy, 2021 https://doi.org/10.3390/antibiotics10091089
97. Shanmugakani, 2020 https://doi.org/10.1039/D0LC00034E
98. Yi, 2020 https://doi.org/10.1021/acsinfecdis.9b00260
99. Brown, 2020 https://doi.org/10.3390/v12060631

Additional studies identified from the references of the 99 included studies (n=27)

1. Xu, 2016 https://doi.org/10.1007/s11033-016-4062-3
2. The European Committee on Antimicrobial Susceptibility Testing. Breakpoint tables for interpretation of MICs and zone diameters https://www.eucast.org/fileadmin/src/media/PDFs/EUCAST_files/Breakpoint_tables/v_11.0_Breakpoint_Tables.pdf
3. CLSI. Performance Standards for Antimicrobial Susceptibility Testing. 30th ed. CLSI supplement M100. Wayne, PA: Clinical and Laboratory Standards Institute; 2020. https://www.nih.org.pk/wp-content/uploads/2021/02/CLSI-2020.pdf
4. EUCAST reading guide 2021 https://www.eucast.org/fileadmin/src/media/PDFs/EUCAST_files/MIC_testing/Reading_guide_BMD_v_3.0_2021.pdf
5. Perillaud, 2019 https://doi.org/10.1016/j.diagmicrobio.2018.07.016
6. Bauer, 1966 https://doi.org/10.1093/ajcp/45.4_ts.493
7. Jorgensen, 2009 https://doi.org/10.1086/647952
8. Eucast, 2019 https://www.eucast.org/fileadmin/src/media/PDFs/EUCAST_files/RAST/EUCAST_RAST_methodology_v1.1_Final.pdf
9. Idelevich, 2018 https://doi.org/10.1016/j.cmi.2017.10.016
10. Garcia-Alvarez, 2011 https://doi.org/10.1016/S1473-3099(11)70126-8
11. Dupieux, 2017 https://doi.org/10.1128/JCM.00068-17
12. Burckart, 2018 https://doi.org/10.3389/fmicb.2018.01744
13. Pancholi, 2018 https://doi.org/10.1128/JCM.01329-17
14. Lutgring, 2018 https://doi.org/10.1128/JCM.01672-17
15. Becker, 2018 https://doi.org/10.3201/eid2402.171074
16. Schwendener, 2017 http://dx.doi.org/10.1038/srep43797
17. Notomi, 2000 https://doi.org/10.1093/nar/28.12.e63
18. Hendriksen, 2019 https://doi.org/10.3389/fpubh.2019.00242
19. Butler, 2016 https://doi.org/10.1038/nprot.2016.036
20. Liu, 2016 http://dx.doi.org/10.1038/srep23375
21. van Belkum, 2018 https://doi.org/10.3389/fmicb.2018.01437
22. Hiramatsu, 2001 https://doi.org/10.1016/S1473-3099(01)00091-3
23. Becker, 2014 https://doi.org/10.1128/CMR.00109-13
24. Hall, 2016 https://doi.org/10.1128/CMR.00062-05
25. Pavlaki, 2013 https://doi.org/10.1016/j.jgar.2013.06.005
26. Buetti, 2017 http://dx.doi.org/10.1136/bmjopen-2016-013665
27. Urushibara, 2019 https://doi.org/10.1093/jac/dkz406
